# Information Model on pain management for elder adults aged 75 years or older *

**DOI:** 10.1590/1518-8345.7111.4305

**Published:** 2024-10-25

**Authors:** Ana Clara de Brito Cruz, Amália de Fátima Lucena, Aline Tsuma Gaedke Nomura, Miriam de Abreu Almeida

**Affiliations:** ^1^ Universidade Federal do Rio Grande do Sul, Escola de Enfermagem, Porto Alegre, RS, Brazil; ^2^ Scholarship holder at the Fundação de Amparo à Pesquisa do Estado do Rio Grande do Sul (FAPERGS), Brazil; ^3^ Scholarship holder at the Conselho Nacional de Desenvolvimento Científico e Tecnológico (CNPq), Brazil; ^4^ Scholarship holder at the Coordenação de Aperfeiçoamento de Pessoal de Nível Superior (CAPES), Brazil; ^5^ Hospital de Clínicas de Porto Alegre, Serviço de Enfermagem Cardiovascular, Nefrologia e Imagem, Porto Alegre, RS, Brazil

**Keywords:** Pain Management, Health of the Elderly, Nurses Improving Care for Health System Elders, Electronic Health Records, Nursing Informatics, Data Mining

## Abstract

to develop an information model on pain management in hospitalized aged people.

a Big Data retrospective and observational study guided by the Applied Healthcare Data Science Roadmap. The sample included all Electronic Health Records related to pain management in older adults aged at least 75 years old considered vulnerable in the institution and admitted to clinical and surgical units. Data science packages were used in Python ^®^ for data analysis.

a total of 9,635 hospitalizations were found for 4,753 patients, with a mean age of 81 years old and 54% belonging to the female gender. The main reasons for hospitalization were diseases of the circulatory system (n=1,593; 28.6%), neoplasms (n=893; 16%) and diseases of the genitourinary system (n=508; 9.1%). A total of 60 attributes related to pain were identified and organized into groups: current pain, assessment instruments and characteristics, Nursing diagnosis, etiology of the Nursing diagnosis, interventions for relief, consultations to specialties and pain reassessment. The groups were classified into four large panels that constituted the information model.

the information model developed presented an overview of the healthcare reality of pain management in vulnerable aged people, supporting decision-making for pain management in this population segment.

## Highlights

**(1)** The model attributes were classified as: pain assessment, interventions and reassessment.

**(2)** Greater importance is given to pain assessment and pharmacological interventions.

**(3)** There was greater variability in the pain care measures prescribed by nurses.

**(4)** Absence of multidimensional instruments for pain assessment was identified.

**(5)** Pain care interventions were prescribed recurrently in aged people at risk of falls.

## Introduction

 The tendency is to expect that pain will increase with age, whether due to accumulation of musculoskeletal injuries, physical wear out with aging or presence of multimorbidities ^(^ 2022 ^-^ 2020 ^)^ . However, pain in aged people is complex, associated with psychosocial factors along with changes in modulation of this signal that are not yet well understood ^(^ 2022 ^)^ . The usual clinical assessment may not encompass these particularities of older adults, especially in the hospital context ^(^ 2019 ^)^ . 

 Different strategies have been developed by hospital programs to meet the comprehensive needs of hospitalized aged people, mainly the prevention of functional decline ^(^ 2022 ^)^ . Age is a determining factor in the development of disabilities during hospitalization. Patients over the age of 75 are more likely to lose their ability to perform activities of daily living during hospitalization ^(^ 2003 ^)^ . With a comprehensive geriatric assessment, older adults have better chances of surviving hospitalization without experiencing cognitive or functional deterioration ^(^ 2022 ^)^ . 

 From this perspective, researchers from a large university hospital located in southern Brazil developed a care protocol for hospitalized aged people ^(^ 2019 ^)^ . Considering the aging population and operationality of the hospital service, there is a focus on longer-lived aged individuals who are more vulnerable to the risk of suffering functional deterioration during hospitalization ^(^ 2003 ^)^ . Therefore, the institutional protocol ^(^ 2019 ^)^ considers all people over the age of 75 as vulnerable. 

 In this context, pain is a sign also related to the reduction of functional capacity and to quality of life worsening ^(^ 2022 ^-^ 2020 ^,^ 2003 ^)^ , constituting an important aspect to be considered when preparing the care plan for hospitalized aged people. 

 To record the care plan, hospital institutions increasingly use electronic information systems, which end up serving as a promising information source. Secondary records collected in the care practice, or Electronic Health Records (EHRs), contain a large volume of information from health services and are continuously produced by different professionals ^(^ 2021 ^)^ . Thus, one of the potentialities of using EHRs is the analysis of diverse health information linked to data science tools, such as Big Data analysis ^(^ 2021 ^-^ 2020 ^)^ . 

 The term “Big Data” refers to the characteristics of the database used, such as large data volume, rapid accumulation of new information and data variety, due to different formats ^(^ 2020 ^)^ . Big Data analysis methods refer to techniques and methods for analyzing a large number of EHRs. These data are very diverse and difficult to visualize using software programs or traditional research tools, requiring the use of specific knowledge and tools to process them ^(^ 2021 ^-^ 2020 ^)^ . 

 Nurses can employ Big Data analyses to improve work processes sensitive to nursing care, both in the care setting and in health management ^(^ 2021 ^-^ 2021 ^)^ . Such analytical approaches allow the development of “Precision Nursing”, aiming to offer personalized care adapted to each person’s particularities, moving towards predictive health models ^(^ 2021 ^,^ 2020 ^)^ . One of the strategies used in North American health institutions to adapt the organization of EHRs, taking advantage of the potential of using these records, is the development of Information Models (IMs) ^(^ 2017 ^)^ . 

 IMs visually elucidate concepts and attributes present in EHRs, allowing all the information to be viewed and accessed more efficiently, making data more understandable and easing the use of the information collected for clinical and managerial decisions ^(^ 2021 ^,^ 2017 ^)^ . In addition, IMs offer support for the construction of ontologies ^(^ 2017 ^-^ 2019 ^)^ , which are formal models of concepts, properties, relationships and functions between pieces of information in a database. A high-level IM with well-defined concepts and relationships allows interoperability of clinical data across different information systems and health institutions ^(^ 2021 ^,^ 2017 ^)^ . 

 Different IMs related to pain and other IMs on topics sensitive to the nursing practice have already been produced, mainly with secondary data from American hospitals ^(^ 2017 ^)^ . In the Brazilian context, a pain management IM was developed using EHR data from adult patients ^(^ 2021 ^)^ . The Brazilian pain management IM included more than 50,000 patients; however, the wide age range covered in the study (from 18 to 107 years old) hinders identifying pain-related care needs and particularities in specific populations, such as the aged population. 

 Considering that pain is a sign that tends to be more present in the population over 70 years old ^(^ 2020 ^)^ , and which exerts impacts on functional capacity ^(^ 2022 ^-^ 2020 ^)^ , the need to improve knowledge related to pain management in older adults was identified. Constructing an IM on pain management in vulnerable aged people (from 75 years old) offers several potential benefits that meet patients’ qualification and safety, as well as improving nursing care for hospitalized older adults. 

Therefore, the current study aims at developing an information model on pain management in hospitalized aged people, seeking to answer the following guiding question: “How can pain management in vulnerable older adults be observed in EHRs?”.

## Method

### Study design

 This is a retrospective and observational study that utilized secondary data, using the data-driven process to analyze the EHRs. Data-driven is an organizational process in which information mapping is used to direct the planning and decision-making process ^(^ 2017 ^)^ . 

### Study locus

 The study was carried out using electronic health records from the Porto Alegre Clinical Complex Hospital ( *Hospital de Clínicas de Porto Alegre* , HCPA), which is a general and public institution linked to the Federal University of Rio Grande do Sul and characterized as a university hospital ^(^ 2023a ^)^ . Regarding hospital management and health care, HCPA is supported by the use of information technology, mainly represented by the Hospital Management Application electronic system called AGHUse. This system eases management and recording of healthcare and administrative activities, was developed by the institution itself and is currently shared with another six public institutions in the country ^(^ 2023b ^)^ . 

 In HCPA aging is understood as an indicator that determines specific assistance needs based on a Protocol for Hospitalized Aged People ^(^ 2019 ^)^ . Nurses evaluate all older adults over the age of 60 for vulnerability risk using the PRISMA-7 assessment scale ^(^ 2008 ^)^ . All older adults are considered vulnerable from the age of 75; in other words, they are more susceptible to risks of losing mental and functional functionality resulting from hospitalization and are assessed in a multiprofessional way ^(^ 2019 ^)^ . 

 Although the PRISMA-7 instrument ^(^ 2008 ^)^ was developed for aged people at least 85 years old, HCPA uses it in an adapted way to screen older adults who may need periodic multiprofessional monitoring, considering that it is an quick-to-apply and easy-to-understand instrument represented for the assistant teams that carry out the screening. 

 All older adults aged at least 75 years old are monitored by the multiprofessional team, while younger aged people are monitored according to the identification of greater vulnerability according to the PRISMA-7 instrument. This is a management choice made by the institution as a way of optimizing monitoring in the line of care for hospitalized aged people, as there is an increasing demand for the institution’s services by increasingly older people, as was observed in 2023 with a 40% increase in the number of hospitalizations of people aged at least 75 years old when compared to the previous year ^(^ 2024 ^)^ . 

 As for the Nursing Process in the institution, it is computerized and has the Basic Human Needs Nursing theoretical framework ^(^ 2016 ^)^ ; in addition, the electronic system covers all stages of the Nursing Process. The NANDA-International terminology (NANDA-I) ^(^ 2021 ^)^ is used in the Nursing Diagnosis (ND) stage, with adaptations to the structure of the electronic system and the institutional reality. Within the adaptations made, the items expressed in the NANDA-I terminology (such as related factors, defining characteristics, risk factors and at-risk populations) are compiled in the electronic system under the term “Etiology”. 

 The Nursing Interventions are associated with Nursing Diagnoses and are based on the Nursing Interventions Classification (NIC) ^(^ 2020 ^)^ terminology and on literature related to the clinical practice. In AGHUse, Nursing Interventions are referred to with the term “Care”. 

### Period

The study data refer to the period between July 2014 and June 2019. The IM on pain management in vulnerable aged people was developed between February 2022 and March 2023.

### Population and sample

 The study population was made up of all structured EHRs originating from electronic medical records, with clinical and sociodemographic data of adult patients admitted to the HCPA clinical and surgical units. The population represents the EHRs collected in a previous research project with adult patients ^(^ 2021 ^)^ . The sample comprised records related to older adults aged at least 75 years old, accounting for more than 3,000,000 observations in EHRs belonging to 7,453 patients. 

### Data collection

To prepare this study, structured clinical and sociodemographic data were used, recorded in electronic medical records and related to pain management in older adults aged at least 75 years old, considered as more vulnerable by the institution.

 This study used queries to select the EHRs from the hospital’s database. The queries were created by the information technology analysts of the hospital institution where the research was developed, and shared through *Excel* tables with the researchers involved. The unprocessed Excel database referring to the larger research project was transformed into CSV (Comma-Separated Values) with the objective of allowing the use of Big Data techniques, considering the data volume. 

The criteria for constructing the queries were as follows: all structured electronic records of adult patients (over 18 years old) admitted to clinical and surgical units. From this database, a framework was designed to further investigate pain in the elderly population aged at least 75 years old, which makes up the current study.

 Handling of the EHRs was guided by the data science roadmap called Applied Healthcare Data Science Roadmap ^(^ 2020 ^)^ . This methodology encompasses six stages: (1) Understanding the research question; (2) Exploratory data analysis; (3) Data preparation; (4) Analytical Modeling; (5) Assessment or validation; and (6) Implementation or deployment. The current study encompasses the first four stages of the script. 

In this methodology, the variables are obtained by exploring the retrospective database in a programming environment, which allows screening all patients; thus, we evaluated patients aged at least 75 years old found in the database.

 Only structured data are considered in this study, that is, data organized in table format. The tables that assemble the database of older adults aged at least 75 years old and the volume of information obtained can be seen in Table 1 . 

Each column of the tables represents a variable to be explored, and each line represents an observation of the variables present in the tables. The number of patients aged at least 75 years old varies from table to table, considering that some interventions – for example, requesting consultations to specialties – were not requested for all patients. Therefore, to construct the IM, the authors considered the total observations of care measures prescribed for pain, and not the number of observations per patient.

**Figure 1-:**
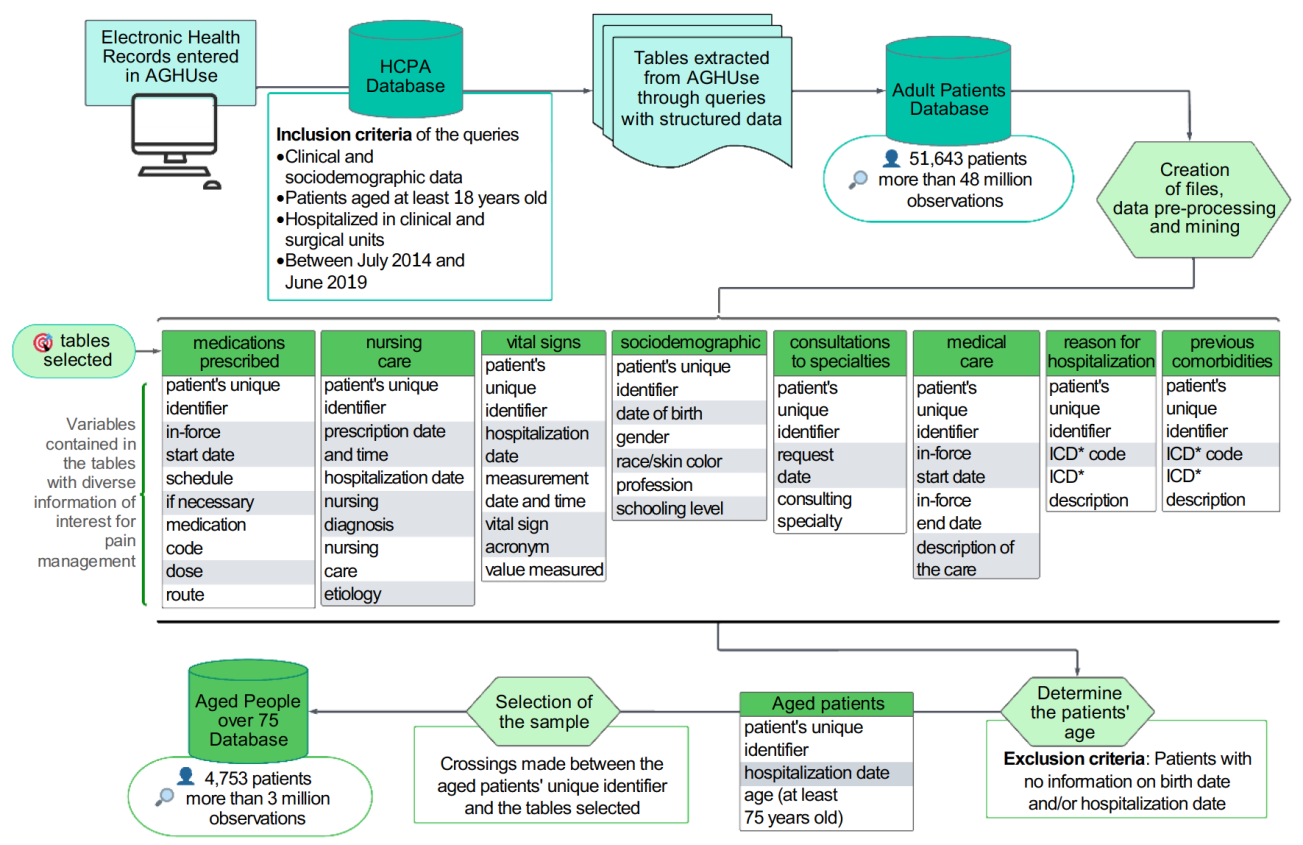
Representation of the collection and preparation of the database that made up the electronic health records of vulnerable aged people

 The representation of data collection in the AGHUse database, the tables and variables used in the database of patients aged at least 75 years old are shown in Figure 1 . 

### Data treatment and analysis

In the treatment and analysis phase, the exploratory analysis stages were carried out: preparation and modeling of data related to pain in patients aged at least 75 years old. Exploratory analysis refers to a broad observation of available data and screening of patients aged 75 or over. When preparing the data, adjustments are made to the database to screen attributes related to pain management, and the attributes found are categorized in the analytical modeling.

### Exploratory data analysis

 An Integrated Development Environment (IDE) was organized to handle the database in the Jupyter notebook in the Python ^®^ programming language and Pandas library packages were used for data analysis ^(^ 2018 ^)^ . 

**Table 1-:** Tables that assemble the database of older adults aged at least 75 years old. Porto Alegre, RS, Brazil, 2019

**Title of the table**	**Number of unique patients**	**Number of variables (columns)**	**Number of observations (lines)**
Consultations to specialties	2,573	5	13,978
Medications prescribed	3,230	10	342,463
Medical care measures	3,291	8	341,531
Parenteral nutrition	44	5	1,142
Previous comorbidities	3,294	3	19,943
Medical procedures	1,596	10	31,644
Reason for hospitalization	1,453	4	5,545
Diet	3,294	10	88,694
Solutions	2,915	9	81,249
Nursing care measures	3,270	15	769,420
Hemotherapy	2,588	6	6,624
Vital signs	2,924	6	1,840,097
Sociodemographic	4,748	6	4,748

*ICD = International Code of Diseases (ICD-10)

 In the IDE, the contents of each variable present in the tables that made up the database were explored in order to identify all the information related to pain management in aged people. Each unique patient had a numerical identifier, herein called “unique patient identifier”, which was used as key identifier ^(^ 2018 ^)^ , with the purpose of crossing the different tables and ensuring that the data refer to patients aged at least 75 years old. 

### Data preparation

During pre-processing, the necessary adjustments were made to the database by excluding data that would enable the identification of the patient or professionals involved in care, as well as the exclusion of incoherent data (for example: typing errors, such as using characters instead of numbers).

To determine the patients’ age, structured data on date of birth associated with the first hospitalization date were considered, as a single patient may have undergone more than one hospitalization during the period under study. Patients with no date of birth and/or no hospitalization date were excluded.

 To mine the data related to pain available in the database, the terms “ *analgesia* ” (“analgesia”), “ doloros* ” (“painful”), “ *dor* ” (“pain”) and “ antálgic* ” (“antalgic”) were searched in all tables, except in the records of prescribed medications, where the medications related to pain were mined by institution identification codes, allowing them to be identified between opioid and non-opioid analgesics. All the information mined was counted by number of observations and data distribution was calculated. In addition to sociodemographic data, data from nursing notes (vital signs and pain levels), Nursing Diagnoses and prescriptions, non-pharmacological medical assistance, medical diagnoses, prescribed medications and medical procedures were used as data sources for developing the IM. 

 Records with less than 50 observations were considered insufficient and disregarded for preparing the IM, according to a previous study ^(^ 2021 ^)^ . The attributes related to pain management in aged people were grouped and categorized. 

### Analytical modeling

 After preparing the database, the diverse information obtained related to pain management in the study population was categorized. This categorization was based on a North American pain management IM ^(^ 2018 ^)^ validated among 10 hospital institutions, considering the institutional cultural characteristics of the study *locus* and the Nursing Process adopted at the institution. 

Based on the categorization, the concepts and attributes were designed with a visual structure of relationships between the results obtained using the LucidChart software. The visual structure developed is the IM on pain management in older adults aged at least 75 years old. This observational modeling allows for a comprehensive understanding of the processes and attributes present in the EHRs and which portray the care practices carried out in the institution.

### Ethical aspects

 To preserve confidentiality and safety of the information, the electronic medical record number of all patients was de-identified by the hospital institution, and the researchers were provided with a numerical identification for each unique patient in the tables. The research project was registered on *Plataforma Brasil* , a national Brazilian institution responsible for research projects ethical evaluation, forwarded to the Research Ethics Committee of the Porto Alegre Clinical Complex Hospital and approved under registration No. 2018.0669. 

## Results

Specific methodologies for Big Data manipulation were used to develop the IM on pain management. Even so, during the exploratory analysis it was necessary to debug and process the data to extract only the information relevant to the study objective.

A total of 4,753 patients were identified, corresponding to 9,635 hospitalizations during the studied period. The mean age of the hospitalized patients was 81 years old, with a maximum of 107. When stratified by age groups, 46.6% of them were between 75 and 79 years old; 30.1% between 80 and 84; 16.7% were between 85 and 89 and 6.6% were aged 90 or over. Among the patients, 2,579 (54.2%) were female and 4,413 (92.8%) were white-skinned. Other racial groups were found in smaller proportions: 250 black-skinned (5.2%), 82 brown-skinned (1.7%) and 08 Asian (0.15%) individuals.

As for schooling level, most of the patients had Incomplete Elementary School (n=2,215; 46.7%), followed by Complete Elementary School (n=854; 17.9%), Complete High School (n=600; 12.7%), Complete Higher Education (n=396; 8.3%), no formal education (n=216; 4.5%), Incomplete High School (n=100; 2.1%) and Incomplete Higher Education (n=36; 0.7%). This record was absent in 336 (7.1%) cases.

The causes for hospital admission in a structured record was found in 5,562 hospitalizations for 1,457 patients. Of this total number of hospitalizations, the causes were grouped into large groups from the International Code of Diseases 10 (ICD-10) to allow better visualization. The main causes recorded were diseases of the circulatory system (n=1,593; 28.6%), neoplasms (n=893; 16%), diseases of the genitourinary system (n=50; 9.1%), diseases of the digestive system (n=504; 9%) and diseases of the respiratory system (n=446; 8%).

When the values were mined to identify the attributes related to pain, 44,093 nursing care measures prescribed for pain were identified, distributed among five main Nursing Diagnoses (NDs) with care prescribed for pain, namely: Acute Pain (73.7%), Chronic Pain (9.5%), Risk of Falls (8.9%), Impaired Comfort (4.8%) and Impaired Physical Mobility (1.7%), in addition to others Nursing Diagnoses that also had care measures prescribed for pain in a lower percentage (1.4%).

The main etiologies related to pain listed for the NDs were: trauma related to an invasive procedure and/or surgical injury (57.3%); disease progression (20%); impaired mobility (7.6%); vascular disturbance (4.4%); biological, chemical, physical and psychological harmful agents (7.6%); disease symptoms or pain (4.2%); and impaired physical mobility (3.6%). It is noted that the “pain” value was present both as an ND and as etiology of a given diagnosis, such as Impaired Physical Mobility. However, the attributes prescribed in Nursing Care were considered for data mining in order to evaluate which NDs had pain related care prescribed more frequently to the population of older adults aged at least 75 years old.

When stratified by ND, the etiologies showed very different distributions. The Acute Pain ND had trauma related to an invasive procedure and/or surgical injury, disease progression and vascular changes as its main etiologies. The Chronic Pain ND presented disease progression and inflammatory process as its main etiologies. When nursing care for pain were prescribed, the Risk of Falls ND presented impaired mobility as its main etiology.

**Figure 2 -:**
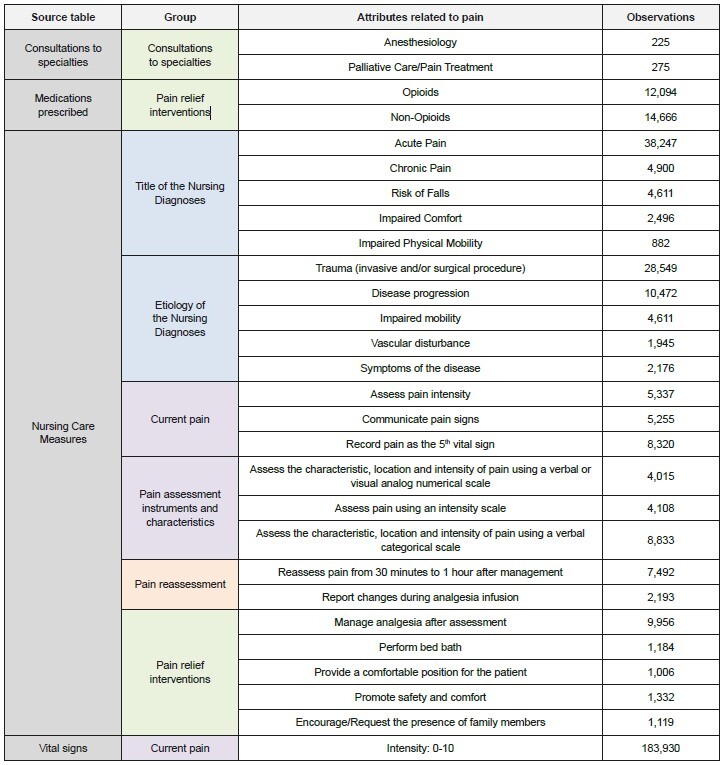
Main attributes found regarding pain management in vulnerable aged people in the electronic health records

 A total of 60 attributes related to pain in vulnerable aged people were identified in different tables in the database; the main attributes in terms of number of observations are described in Figure 2 . The attributes identified were organized into seven groups: (1) Current pain, (2) Pain assessment instruments and characteristics, (3) Title of the Nursing Diagnoses, (4) Etiology of the Nursing Diagnoses, (5) Interventions for pain relief, (6) Consultations with specialties and (7) Pain reassessment. The organization into groups served as support for constructing the conceptual definitions corresponding to the groups of attributes, improving semantic understanding of the IM developed. 

These seven different groups of attributes were grouped and organized into classification panels: (1) Assessment Panel; (2) Intervention Panel; (3) Nursing Diagnosis Panel and their components; and (4) Reassessment Panel.

**Figure 3 -:**
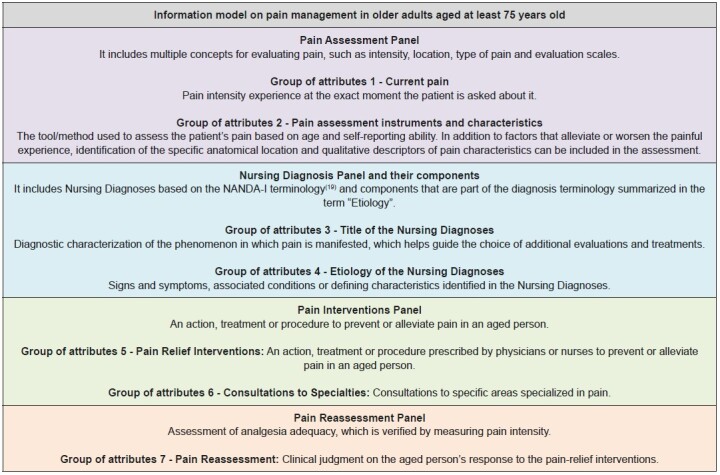
Descriptive definition corresponding to the groups of attributes and to the classification (panels) used in the development of the Information Model

 Classification of the attributes resorted to a validated pain management IM ^(^ 2018 ^)^ as basis, adding aspects related to the institutional culture and the organization flowchart of the hospital information system in its construction. Therefore, the components related to pain management in vulnerable aged people were organized based on the EHRs. For semantic understanding of the IM developed, the conceptual definition of the classification and groups of attributes are described in Figure 3 . 

**Figure 4 -:**
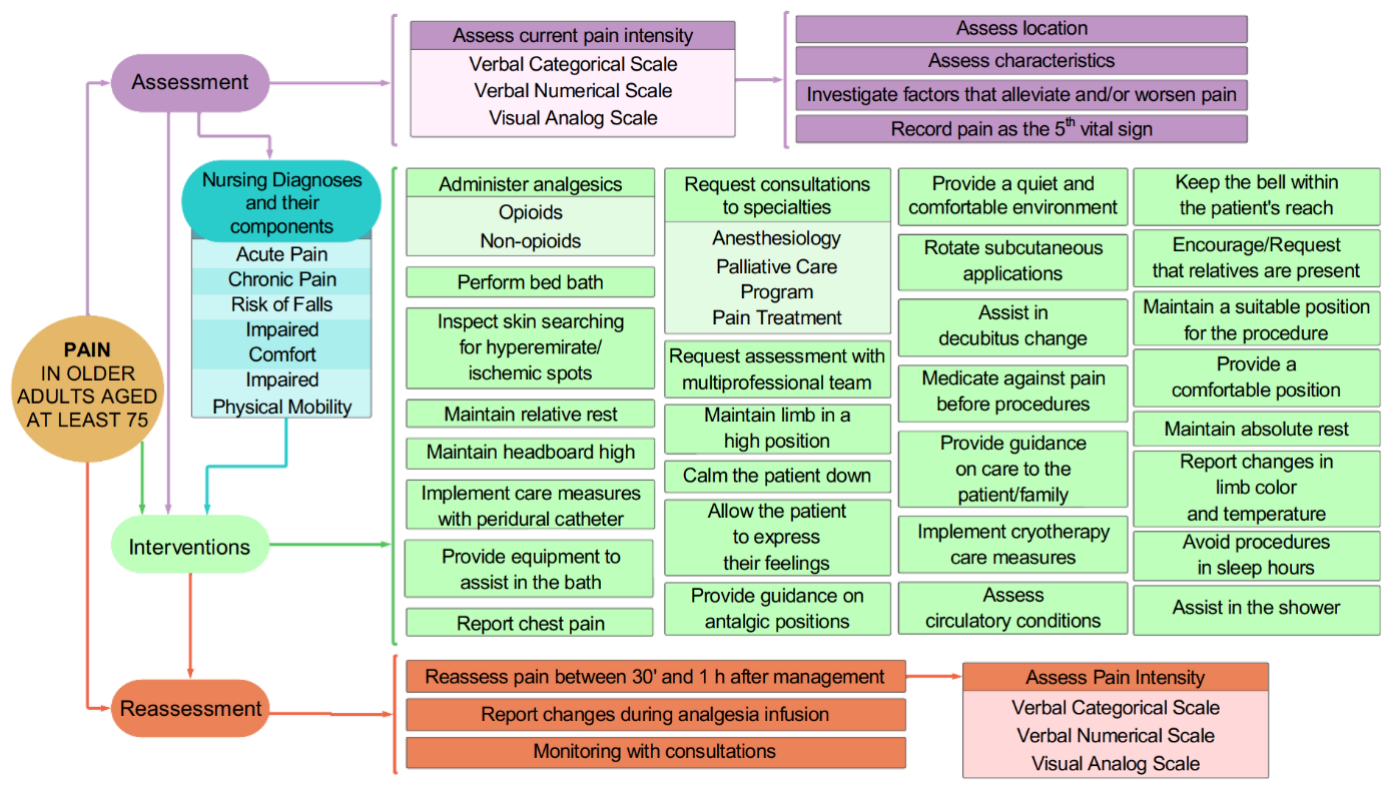
Information Model on pain management in elder adults aged 75 years or older

 Finally, based on the pain-related EHR data that were mined in the study sample and after classifying and organizing these attributes, an IM on pain management in vulnerable aged people at a university hospital in the South of the country was developed. The IM developed is represented in a visual structure in Figure 4 . 

## Discussion

The first IM on pain management in vulnerable aged people was developed from the structured EHRs of older adults over the age of 75. Although the database is only restricted to structured data, mining using data science techniques allowed obtaining extensive information with a significant volume of the sample under study.

 IMs on pain management have already been developed with large databases both in the United States ^(^ 2017 ^)^ and in Brazil ^(^ 2021 ^)^ , but not specifically for aged people. The North American IM on pain management ^(^ 2017 ^)^ , with data from several health institutions, was validated by an experts’ evaluation ^(^ 2018 ^)^ , improving its clinical applicability and offering perspectives for interoperability across health systems. The IM developed in the current study was prepared with data from a single hospital institution, which reduces its potential for generalization. 

 Age stratification between aged and vulnerable aged patients (at least 75 years old) is a tool for operationalizing the monitoring of older adults hospitalized in the institution ^(^ 2019 ^)^ . However, there is controversy in the literature regarding the relevance of age in the prevalence of pain ^(^ 2018 ^)^ . Age was not observed as a significantly different factor between aged individuals with and without body pain ^(^ 25 ^)^ , in knee pain ^(^ 2020 ^-^ 2023 ^)^ . However, age is a significant factor in pain intensity in patients with low back pain ^(^ 2023 ^)^ , after total hip arthroplasty ^(^ 2022 ^)^ and in signs of changes in pain perception after a stroke ^(^ 2022 ^)^ . 

 Such scenarios evidence the complexity of pain in aged people, justifying the development of IMs that evaluate care measures related to pain management in specific groups. The IM developed in this study allowed classifying the attributes mined for pain in pain assessment, interventions and reassessment. These four major pain management classification panels at the institution originated from the seven groups of pain-related attributes, based on the validated American IM ^(^ 2017 ^)^ . 

 The use of unidimensional instruments (which only allow assessing intensity) in pain assessment ^(^ 2019 ^)^ is observed in the EHRs ^(^ 2019 ^)^ , such as visual analogue, verbal numerical and verbal categorical scales. In general, unidimensional scales are good instruments for assessing pain and present easy use in the clinical practice ^(^ 2018 ^-^ 2018 ^)^ ; however, in the case of aged people, these tools may not be sufficient due to cognitive disabilities, difficulties in communicating the painful experience and behavioral factors ^(^ 2018 ^,^ 2019 ^)^ . 

 Multidimensional pain assessment instruments allow better knowledge about the psychological, social and economic aspects that impact the painful experience and also support a more robust anamnesis for older adults ^(^ 2019 ^)^ . There are five multidimensional pain assessment instruments validated in Brazil ^(^ 2016 ^-^ 2006 ^)^ ; some of them are adapted for aged people with limited communication capacity or for non-communicative patients ^(^ 2016 ^-^ 2012 ^)^ , in addition to specific instruments for older adults with dementia ^(^ 2016 ^)^ . None of these instruments is used in the institution, which constitutes a weakness in comprehensive health care for aged people. 

 In the EHRs, greater variability was observed in the pain-related care measures prescribed by nurses, encompassing physiological, psychological and social aspects involved in pain management. Despite this, the volume of observations indicates greater importance given to assessment and pharmacological interventions for pain, similarly to what is found in another Brazilian study ^(^ 2020 ^)^ . IMs offer support in this sense, allowing understanding the relationships and complexities of the flow of information on topics of interest to the Nursing practice ^(^ 2021 ^,^ 2017 ^)^ . 

When constructing the IM it was decided to include the NDs and their components as one of the groups represented due to the large number of structured records, which represent institutional appreciation regarding application of the Nursing Process. Presence of three NDs with prescribed care for pain in addition to those that are specific (Acute Pain ND and Chronic Pain ND) was included in the IM developed, namely: Risk of Falls, Impaired Comfort and Impaired Physical Mobility.

 These three NDs refer to impairments in motor functionality and physical mobility, common events in aged individuals, especially those hospitalized ^(^ 2021 ^-^ 2021 ^)^ . It is important to note that nurses diagnose health problems and risk conditions based on a human response that may or may not be associated with other medical conditions ^(^ 2021 ^)^ . Therefore, the mobility impairment that leads nurses to list the Risk of Falls, Impaired Comfort or Impaired Physical Mobility NDs identifies this sign as a need for care at that moment. Both human responses and NDs are dynamic, reflecting a need at the time they were assessed, and may have started with this demand before or during hospitalization. 

 As for the elaboration of our IM on pain management, we mined values present in the care measures prescribed, the data found in this analysis suggest that pain signs and symptoms can be associated with a greater risk of falls in vulnerable aged people, considering the recurrence of pain care measures prescribed in the Risk of Falls ND – the third ND with the highest volume of pain care measures prescribed. This feature is not present in the Brazilian IM on pain management developed with adult patients ^(^ 2021 ^)^ . 

 Different studies ^(^ 2021 ^-^ 2021 ^)^ indicate that the risk of falls was not found to be a variable associated with pain, regardless of its intensity; nor as a protective factor against the occurrence of falls ^(^ 2020 ^)^ . On the other hand, 

 a study carried out with middle-aged and older adults identified presence of pain signs as a factor that increases the risk of falls ^(^ 2020 ^)^ , as well as a study ^(^ 2021 ^)^ carried out with older people aged at least 70 years old. Due to the descriptive characteristic of the IM developed, it is not possible to assert whether there is an association between pain and risk of falls, but more detailed analyses of this relationship represent a gap that needs to be further explored. 

 There is strong evidence that a smaller support network for aged people is related both to the risk of falls and to worse pain management ^(^ 2022 ^)^ , in addition to being a factor related to functional capacity in this population group ^(^ 2020 ^)^ . In itself, this justifies screening and monitoring protocols for these patients who must be evaluated in a multidimensional way, aiming to organize the support network in planning their discharge. 

This study had limitations in its design that need to be considered when evaluating the results presented. The IM developed only relied on structured data from the patients’ electronic medical records for its construction, excluding documents considered important such as Nursing evolution/anamnesis and physical examination, as they are free-text records.

As this study resorted to secondary records, it is also subjected to information bias, such as recording errors or missing data like the patients’ hospitalization date, which was used to later determine the age of the sample. To address this limitation, this variable was treated in such a way as to consider the first occurrence of a nursing or medical prescription recorded as the patients’ admission date.

Another important limitation was mapping through exploratory analysis and construction of programming commands only by one researcher, which is subjected to errors when extracting information from the database. In addition to that, the attributes on pain management in vulnerable aged people were mapped at a single institution and in medical records of patients admitted to clinical and surgical units.

 Regarding the health management and research aspects, IMs are the initial component in constructing semantic models that ground interface projects of health information systems. Both the IM developed in this study and the one on pain management in adults developed in Brazil ^(^ 2021 ^)^ were constructed based on data from a single institution. In future proposals, they can serve as support for data interoperability between more than one institution, supporting the construction of data-driven assistance with much greater generalization potential. 

## Conclusion

The IM allowed obtaining an overview of the pain management reality in hospitalized older adults aged at least 75 years old. Development of the IM made it possible to organize records related to pain management and classifying them into large groups that reflect the care provided to aged people, as well as the institutional resources used to manage pain in this population segment.

Institutional maturity is observed regarding application of the Nursing Process in a structured manner, in addition to presence of different types of nursing interventions that encompass biological, psychological and psychosocial aspects inherent to nursing care.

Regarding the care gaps observed, absence of multidimensional instruments for pain management in the records is identified, which makes it difficult to identify this sign in aged people with functional impairment or deficits in understanding and communicating pain through numerical scales.

The study allowed observing that physical mobility impairment appears as a prevalent factor in the records of pain care measures prescribed for vulnerable aged people. It is deduced that presence of pain combined with impaired mobility in older adults may increase the risk of fall episodes. However, studies with more robust designs that allow evaluating this association would need to be conducted.

The IM developed has not been validated. In a prospective scenario, validation and implementation of the IM can serve as support for semantic interoperability between information systems or in the development of predictive models on pain management, especially for the aged population; in addition to optimizing hospital information systems.
