# Supplementary material for: Information Model on pain management for elder adults aged 75 years or older
Source: Rev Lat Am Enfermagem. 2024 Oct 25;32:e4305. doi: 10.1590/1518-8345.7111.4305 (PMC11526214; doi:10.1590/1518-8345.7111.4305)
Supplement: Translation

# Translation: Modelo de información del manejo del dolor en adultos mayores con 75 años o más*

**DOI:** 10.1590/1518-8345.7111.4304

**Published:** 2024-10-09

**Authors:** Ana Clara de Brito Cruz, Amália de Fátima Lucena, Aline Tsuma Gaedke Nomura, Miriam de Abreu Almeida

**Affiliations:** ^1^ Universidade Federal do Rio Grande do Sul, Escola de Enfermagem, Porto Alegre, RS, Brasil; ^2^ Becaria de la Fundação de Amparo à Pesquisa do Estado do Rio Grande do Sul (FAPERGS), Brasil; ^3^ Becaria del Conselho Nacional de Desenvolvimento Científico e Tecnológico (CNPq), Brasil; ^4^ Becaria de la Coordenação de Aperfeiçoamento de Pessoal de Nível Superior (CAPES), Brasil; ^5^ Hospital de Clínicas de Porto Alegre, Serviço de Enfermagem Cardiovascular, Nefrologia e Imagem, Porto Alegre, RS, Brasil

**Keywords:** Manejo del Dolor, Salud del Anciano, Enfermería para la Mejora de la Atención Sanitaria Geriátrica, Registros Electrónicos de Salud, Informática Aplicada a la Enfermería, Minería de Datos

## Abstract

desarrollar un modelo de información sobre el manejo del dolor en adultos mayores hospitalizados.

estudio observacional retrospectivo de *Big Data* guiado por el *Applied Healthcare Data Science Roadmap* . La muestra incluyó todos los Registros Médicos Electrónicos relacionados con el manejo del dolor en adultos mayores de 75 años o más, considerados vulnerables en la institución, ingresados en unidades clínicas y quirúrgicas. Para el análisis se utilizaron paquetes de ciencia de datos en Python ^®^ .

se encontraron 9.635 hospitalizaciones para 4.753 pacientes, con edad promedio de 81 años, de los cuales 54% eran del sexo femenino. Los principales motivos de hospitalización fueron enfermedades del sistema circulatorio (n=1.593, 28,6%), neoplasias (n=893, 16%) y enfermedades del sistema genitourinario (n=508, 9,1%). Se identificaron 60 atributos relacionados con el dolor, organizados en los siguientes grupos: dolor actual, instrumentos y características de evaluación, diagnóstico de enfermería, etiología del diagnóstico de enfermería, intervenciones para el alivio, consultoría de especialidades y reevaluación del dolor. Los grupos se clasificaron en cuatro grandes paneles que conformaron el modelo de información.

el modelo de información desarrollado presentó una visión general de la realidad asistencial del manejo del dolor en adultos mayores vulnerables, que contribuye a la toma de decisiones para el manejo del dolor en esta población.

## Destacados

**(1)** Los atributos se clasificaron en: evaluación, intervenciones y reevaluación del dolor.

**(2)** Se les da mayor importancia a la evaluación y a las intervenciones farmacológicas del dolor.

**(3)** Hubo mayor variabilidad en los cuidados para el dolor prescritos por los enfermeros.

**(4)** Se identificó que no había instrumentos multidimensionales para evaluar el dolor.

**(5)** Se prescribieron cuidados para el dolor de forma recurrente en ancianos con riesgo de caídas.

## Introducción

 Tendemos a esperar que el dolor aumente con la edad, ya sea por el incremento de las lesiones musculoesqueléticas, el desgaste físico con el envejecimiento o la presencia de multimorbilidades ^(^ 2022 ^-^ 2020 ^)^ . Sin embargo, el dolor en los adultos mayores es complejo, se asocia con factores psicosociales junto con cambios en la modulación de esta señal que aún no están claros ^(^ 2022 ^)^ . La evaluación clínica habitual puede no incluir esas particularidades de los adultos mayores, especialmente en el contexto hospitalario ^(^ 2019 ^)^ . 

 Los programas hospitalarios desarrollaron diferentes estrategias para satisfacer las necesidades integrales de las personas ancianas hospitalizadas, principalmente la prevención del deterioro funcional ^(^ 2022 ^)^ . La edad es un factor determinante en el desarrollo de discapacidad durante la hospitalización. Los pacientes mayores de 75 años tienen más probabilidades de perder la capacidad de realizar actividades de la vida diaria durante la hospitalización ^(^ 2003 ^)^ . Con una evaluación geriátrica integral, los adultos mayores tienen mayores posibilidades de sobrevivir a la hospitalización sin sufrir deterioro cognitivo o funcional ^(^ 2022 ^)^ . 

 Debido a ello, investigadores de un gran hospital universitario, ubicado en el sur de Brasil, desarrollaron un protocolo de atención para adultos mayores hospitalizados ^(^ 2019 ^)^ . Considerando el envejecimiento poblacional y la operatividad de los servicios hospitalarios, se centró en los ancianos de edad más avanzada, que son más vulnerables al riesgo de sufrir deterioro funcional durante la hospitalización ^(^ 2003 ^)^ . Por ende, el protocolo institucional ^(^ 2019 ^)^ considera vulnerables a todos los ancianos mayores de 75 años. 

 El dolor, en este contexto, es un signo que se relaciona también con la reducción de la capacidad funcional y el empeoramiento de la calidad de vida ^(^ 2022 ^-^ 2020 ^,^ 2003 ^)^ , y es un aspecto importante que hay que considerar a la hora de elaborar el plan de cuidados de los adultos mayores hospitalizados. 

 Para registrar el plan de cuidados, las instituciones hospitalarias utilizan cada vez más sistemas de información electrónicos; que en definitiva son una fuente de información prometedora. Los registros secundarios recopilados en la práctica asistencial, o Registros Electrónicos de Salud (RES), contienen un gran volumen de información proveniente de los servicios de salud y son producidos continuamente por diferentes profesionales ^(^ 2021 ^)^ . Por ende, uno de los posibles usos de los RES es el análisis de información de salud vinculado a herramientas de ciencia de datos, como el análisis de datos *Big Data* ^(^ 2021 ^-^ 2020 ^)^ . 

 El término *Big Data* hace referencia a las características de la base de datos utilizada, tales como gran volumen de datos, rápida acumulación de nueva información y variedad de datos, debido a los diferentes formatos ^(^ 2020 ^)^ . Los métodos de análisis de *Big Data* implican técnicas y métodos para analizar una gran cantidad de RES. Estos datos son muy diversos y difíciles de visualizar utilizando *software* o herramientas tradicionales de investigación y requieren el uso de conocimientos y herramientas específicos para procesarlos ^(^ 2021 ^-^ 2020 ^)^ . 

 Los análisis de *Big Data* pueden ser utilizados por los enfermeros para mejorar los procesos de trabajo sensibles para los cuidados de enfermería, tanto en el ámbito asistencial como en la gestión en salud ^(^ 2021 ^-^ 2021 ^)^ . Esos enfoques analíticos permiten el desarrollo de una “enfermería de precisión”, con el fin de brindar una atención personalizada, adaptada a las particularidades de cada individuo, y dirigida a modelos predictivos de salud ^(^ 2021 ^,^ 2020 ^)^ . Una de las estrategias utilizadas en las instituciones de salud estadounidenses para adaptar la organización de los RES, aprovechando el potencial de uso de estos registros, es la elaboración de Modelos de Información (MI) ^(^ 2017 ^)^ . 

 Los MI aclaran visualmente conceptos y atributos presentes en los RES, lo que permite ver y acceder a la información de manera más eficiente, y hace que los datos sean más comprensibles y facilita el uso de la información recopilada para tomar decisiones clínicas y de gestión ^(^ 2021 ^,^ 2017 ^)^ . Además, los MI brindan soporte para la construcción de ontologías ^(^ 2017 ^-^ 2019 ^)^ , que son un modelo formal de conceptos, propiedades, relaciones y funciones entre la información de una base de datos. Un MI de alto nivel, con conceptos y relaciones bien definidas, permite la interoperabilidad de datos clínicos entre diferentes sistemas de información e instituciones de salud ^(^ 2021 ^,^ 2017 ^)^ . 

 Ya se han producido diferentes MI relacionados con el dolor y otros MI sobre temas sensibles a la práctica de enfermería, principalmente con datos secundarios de hospitales estadounidenses ^(^ 2017 ^)^ . En el contexto brasileño, se desarrolló un MI para el manejo del dolor utilizando datos de RES de pacientes adultos ^(^ 2021 ^)^ . El MI brasileño de manejo del dolor incluyó más de 50 mil pacientes, pero el amplio rango etario que abarca el estudio, de 18 a 107 años, dificulta identificar las necesidades de atención y particularidades relacionadas con el dolor de ciertas poblaciones específicas, como la de personas mayores. 

 Dado que el dolor es un signo que tiende a estar más presente en la población mayor de 70 años ^(^ 2020 ^)^ y que es un signo que afecta la capacidad funcional ^(^ 2022 ^-^ 2020 ^)^ , se identificó que era necesario mejorar los conocimientos relacionados con el manejo del dolor en los adultos mayores vulnerables. La construcción de un MI para el manejo del dolor en los adultos mayores vulnerables de 75 años o más, tiene varios beneficios potenciales para lograr la calificación y seguridad del paciente; así como para mejorar los cuidados de enfermería para los adultos mayores hospitalizados. 

Por lo tanto, el presente estudio tiene como objetivo desarrollar un modelo de información sobre el manejo del dolor en los adultos mayores hospitalizados, y busca responder a la pregunta orientadora: “¿Cómo se puede observar en el RES el manejo del dolor en los adultos mayores vulnerables?”.

## Método

### Diseño del estudio

 Este es un estudio observacional retrospectivo, que utiliza datos secundarios, realizado mediante el proceso de *data-driven* para analizar los RES. *Data-driven* es un proceso organizacional en el que se utiliza el mapeo de información para conducir la planificación y el proceso de toma de decisiones ^(^ 2017 ^)^ . 

### Lugar del estudio

 El estudio se realizó utilizando Registros Médicos Electrónicos del Hospital de Clínicas de Porto Alegre (HCPA), que es general y público, vinculado a la Universidad Federal de Rio Grande do Sul, por lo que es un hospital escuela ^(^ 2023a ^)^ . La gestión hospitalaria y la atención de salud del HCPA se basan en el uso de tecnologías de la información, principalmente de la Aplicación de Gestión Hospitalaria, sistema de información denominado AGHUse. Este sistema, que facilita la gestión y el registro de las actividades asistenciales y administrativas, fue desarrollado por la misma institución y actualmente lo comparten otras seis instituciones públicas del país ^(^ 2023b ^)^ . 

 En el HCPA, el envejecimiento se considera un indicador que determina las necesidades asistenciales específicas y se basa en un Protocolo de Atención para el Anciano Hospitalizado ^(^ 2019 ^)^ . Los enfermeros evalúan a todos los adultos mayores de 60 años o más para detectar el riesgo de vulnerabilidad mediante la escala de evaluación PRISMA-7 ^(^ 2008 ^)^ . A partir de los 75 años, todos los adultos mayores son considerados vulnerables, es decir, son más susceptibles a riesgos de pérdida de funcionalidad mental y funcional derivadas de la hospitalización y son evaluados de forma interdisciplinaria ^(^ 2019 ^)^ . 

 Si bien el instrumento PRISMA-7 ^(^ 2008 ^)^ fue elaborado para adultos mayores de 85 años o más, el HCPA lo utiliza de forma adaptada para monitorear a los adultos mayores que pueden necesitar un seguimiento interdisciplinario periódico, dado que es un instrumento de rápida aplicación y fácil de entender para los equipos de salud que realizan el seguimiento. 

 El equipo interdisciplinario realiza el seguimiento de todos los adultos mayores de 75 años o más y de los más jóvenes considerados con mayor vulnerabilidad según el instrumento PRISMA-7. Esta elección de gestión que realiza la institución es una forma de optimizar el seguimiento en la línea de atención a los adultos mayores hospitalizados, dada la creciente demanda de los servicios de la institución por parte de personas que tienen cada vez más edad, como se puede ver en los resultados de 2023, que hubo un aumento del 40% en el número de hospitalizaciones de personas de 75 años o más con respecto al año anterior ^(^ 2024 ^)^ . 

 En la institución del estudio el Proceso de Enfermería es informatizado y el marco teórico es el de las Necesidades Humanas Básicas ^(^ 2016 ^)^ , y el sistema electrónico incluye todas las etapas del proceso de enfermería. En la etapa de diagnóstico de enfermería (DE), se utiliza la terminología de la asociación NANDA-Internacional (NANDA-I) ^(^ 2021 ^)^ , con adaptaciones a la estructura del sistema electrónico y a la realidad institucional. Dentro de las adaptaciones realizadas, los ítems expresados en la terminología NANDA-I como: factores relacionados, características definitorias, factores de riesgo y poblaciones en riesgo se recopilan en el sistema electrónico bajo el término “etiología”. 

 Las intervenciones de enfermería están asociadas a los diagnósticos de enfermería y se basan en la terminología de la Clasificación de Intervenciones de Enfermería ( *Nursing Interventions Classification* , NIC) ^(^ 2020 ^)^ y en la literatura relacionada con la práctica clínica. En AGHUse, las intervenciones de enfermería se denominan “cuidados”. 

### Periodo

Los datos del estudio se refieren al periodo comprendido entre julio de 2014 y junio de 2019. El desarrollo del MI para el manejo del dolor en los adultos mayores vulnerables se llevó a cabo entre febrero de 2022 y marzo de 2023.

### Población y muestra

 La población del estudio estuvo compuesta por todos los RES estructurados, provenientes de historias clínicas electrónicas, con datos clínicos y sociodemográficos de pacientes adultos ingresados en las unidades clínicas y quirúrgicas del HCPA. La población representa los RES recolectados en un proyecto de investigación anterior con pacientes adultos ^(^ 2021 ^)^ . La muestra estuvo compuesta por registros de adultos mayores de 75 años o más; hay más de tres millones de observaciones en los RES que pertenecen a 7.453 pacientes. 

### Recolección de datos

Para realizar el estudio se utilizaron datos clínicos y sociodemográficos estructurados, registrados en historias clínicas electrónicas, relacionados con el manejo del dolor en los adultos mayores de 75 años o más, que la institución considera más vulnerables.

 Este estudio utilizó *queries* para seleccionar RES de la base de datos del hospital. Las *queries* fueron creadas por los analistas de tecnologías de la información de la institución hospitalaria en la que se desarrolló la investigación y compartidas con los investigadores involucrados a través de tablas de *Excel* . La base de datos en bruto de *Excel* , relativa al proyecto de investigación más amplio, se transformó en CSV ( *Comma-Separated Values* ) para poder usar técnicas de manejo de *Big Data* , debido al volumen de los datos. 

 Los criterios para crear las *queries* fueron los siguientes: todos los registros electrónicos estructurados de pacientes adultos (mayores de 18 años), ingresados en unidades clínicas y quirúrgicas. A partir de esta base de datos, se realizó un recorte para investigar más a fondo el dolor en la población de edad avanzada de 75 años o más, que es la que conforma el presente estudio. 

 El manejo de los RES se basó en la guía de ciencia de datos denominada *Applied Healthcare Data Science Roadmap* ^(^ 2020 ^)^ . Esta metodología incluye seis pasos: (1) Comprender la pregunta de investigación; (2) Análisis exploratorio de los datos; (3) Preparación de los datos; (4) Modelado Analítico; (5) Evaluación o validación; (6) Implementación o despliegue. El presente estudio abarca las primeras cuatro etapas de la guía. 

En esta metodología las variables se obtienen explorando la base de datos retrospectiva en un entorno de programación, lo que permite el seguimiento de todos los pacientes; de esa forma, evaluamos a los pacientes de 75 años o más que se encuentran en la base de datos.

 En este estudio solo se consideran datos estructurados, es decir, datos organizados en formato de tabla. Las tablas que componen la base de datos de los adultos mayores de 75 años o más y el volumen de información obtenida se pueden consultar en la Tabla 1 . 

Cada columna de las tablas representa una variable a explorar y cada línea representa una observación sobre las variables presentes en las tablas. El número de pacientes de 75 años o más varía de una tabla a otra, considerando que algunas intervenciones, por ejemplo, solicitar consultoría de especialidades, no fueron solicitadas para todos los pacientes. Por lo tanto, para desarrollar el MI, los autores consideraron el total de las observaciones de los cuidados prescritos para el dolor, y no el número de observaciones por paciente.

### Tratamiento y análisis de los datos

En el tratamiento y análisis se realizaron los pasos de análisis exploratorio; preparación y modelado de datos relacionados con el dolor en pacientes de 75 años o más. El análisis exploratorio implica una observación amplia de los datos disponibles y seguimiento de los pacientes de 75 años o más.

**Tabla 1:** - Tablas que componen la base de datos de los adultos mayores de 75 años o más. Porto Alegre, RS, Brasil, 2019

**Título de la tabla**	**Nº de pacientes únicos**	**Nº de variables (columnas)**	**Nº de observaciones (líneas)**
Consultoría de especialidades	2.573	5	13.978
Medicamentos prescritos	3.230	10	342.463
Cuidados médicos	3.291	8	341.531
Nutrición Parenteral	44	5	1.142
Comorbilidades previas	3.294	3	19.943
Procedimientos médicos	1.596	10	31.644
Motivo de la internación	1.453	4	5.545
Dieta	3.294	10	88.694
Soluciones	2.915	9	81.249
Cuidados de enfermería	3.270	15	769.420
Hemoterapia	2.588	6	6.624
Signos vitales	2.924	6	1.840.097
Sociodemográficos	4.748	6	4.748

**Figura 1 -:** Representación de la recolección y preparación de la base de datos que conformaron los Registros Médicos Electrónicos de los adultos mayores vulnerables

*CIE = Clasificación Internacional de Enfermedades (CIE-10)

Al preparar los datos, se realizan ajustes en la base de datos para detectar los atributos relacionados con el manejo del dolor; y en el modelado analítico, se categorizaron los atributos encontrados.

### Análisis exploratorio de los datos

 Para el manejo de la base de datos se organizó un *Integrated Development Environment* (IDE) en el Jupyter *notebook* en lenguaje de programación Python ^®^ y para el análisis de los datos se utilizaron paquetes de la biblioteca Pandas ^(^ 2018 ^)^ . 

 En el IDE se exploró el contenido de cada variable presente en las tablas que conformaron la base de datos con el objetivo de identificar información relacionada con el manejo del dolor en los adultos mayores. Cada paciente único contaba con un identificador numérico, que se denominó “identificador único del paciente” y se utilizaba como identificador clave ^(^ 2018 ^)^ , con el fin de realizar un cruzamiento entre las diferentes tablas y garantizar que los datos correspondieran a pacientes de 75 años o más. 

### Preparación de los datos

Durante el preprocesamiento se realizaron los ajustes necesarios a la base de datos, se excluyeron los datos que permitían identificar al paciente o a los profesionales involucrados en la atención y los datos incoherentes (por ejemplo: errores de tipeo, el uso de caracteres en lugar de números).

Para determinar la edad de los pacientes se consideraron datos estructurados sobre fecha de nacimiento asociada a la fecha de la primera hospitalización; dado que un solo paciente puede haber tenido más de una hospitalización durante el período estudiado. Se excluyeron los pacientes sin fecha de nacimiento y/o sin fecha de hospitalización.

Para extraer los datos relacionados con el dolor disponibles en la base de datos, se buscaron los términos “analgesia”, “doloros*”, “dolor” y “antálgic*” en todas las tablas, excepto en los registros de medicamentos prescritos, dado que los medicamentos relacionados al dolor fueron extraídos por códigos de identificación institucional, lo que permitió distinguir entre analgésicos opioides y no opioides. La información extraída se contabilizó por número de observaciones y se calculó la distribución de datos. Como fuentes de datos para elaborar el MI se utilizaron datos de las notas de los enfermeros (signos vitales y niveles de dolor), diagnósticos y prescripciones de los enfermeros, asistencia médica no farmacológica, diagnósticos médicos, medicamentos prescritos, procedimientos médicos, además de datos sociodemográficos.

 Los registros con menos de 50 observaciones fueron considerados insuficientes y descartados para la elaboración del MI, según un estudio anterior ^(^ 2021 ^)^ . Se agruparon y categorizaron los atributos relacionados con el manejo del dolor en los adultos mayores. 

### Modelado analítico

 Luego de elaborar la base de datos, se categorizó la información obtenida sobre el manejo del dolor en la población de estudio. La categorización se basó en un MI estadounidense sobre manejo del dolor ^(^ 2018 ^)^ validado entre 10 instituciones hospitalarias; se consideraron las características culturales institucionales del lugar del estudio y del proceso de enfermería adoptado en la institución. 

 A partir de la categorización, se diseñaron los conceptos y atributos con una estructura visual de las relaciones entre los resultados obtenidos mediante el *software* *LucidChart* . La estructura visual desarrollada es el MI para el manejo del dolor en adultos mayores de 75 años o más. Este modelado observacional permite tener una comprensión integral de los procesos y atributos presentes en los RES y que reflejan las prácticas de cuidado que se realizan en la institución. 

### Aspectos éticos

Para mantener la confidencialidad y la seguridad de la información, la institución hospitalaria anonimizó el número de la historia clínica electrónica de todos los pacientes y les dio a las investigadoras una identificación numérica para cada paciente único en las tablas. El proyecto de investigación fue registrado en Plataforma Brasil, enviado al Comité de Ética en Investigación del Hospital de Clínicas de Porto Alegre y aprobado bajo registro n.º 2018.0669.

## Resultados

 Para desarrollar el MI sobre el manejo del dolor, se utilizaron metodologías específicas para el manejo de *Big Data* . Aun así, durante el análisis exploratorio fue necesario limpiar y procesar los datos para extraer sólo la información relevante para el objetivo del estudio. 

Se identificaron 4.753 pacientes, correspondientes a 9.635 hospitalizaciones durante el período estudiado. La edad promedio de los pacientes hospitalizados fue de 81 años, el mayor tenía 107 años. Al estratificarlos por franja etaria, el 46,6% de ellos tenía entre 75 y 79 años; el 30,1% entre 80 y 84 años; el 16,7% entre 85 y 89 años y el 6,6% 90 años o más. De los 4.753 pacientes, 2.579 (54,2%) eran del sexo femenino y 4.413 (92,8%) eran blancos. Se observaron otros grupos raciales en menor proporción: 250 negros (5,2%), 82 morenos (1,7%) y 08 asiáticos (0,15%).

En cuanto al nivel educativo, la mayoría de los pacientes tenía educación básica incompleta (n=2.215, 46,7%), seguido de educación básica completa (n=854, 17,9%), educación secundaria completa (n=600, 12,7%), educación superior completa (n=396, 8,3%), sin educación formal (n=216, 4,5%), educación secundaria incompleta (n=100, 2,1%), educación superior incompleta (n=36, 0,7%). El nivel educativo no fue registrado en 336 (7,1%) casos.

El motivo de hospitalización en el registro estructurado figuraba en 5.562 hospitalizaciones de 1.457 pacientes. Las causas del número total de hospitalizaciones se agruparon en grandes grupos de la Clasificación Internacional de Enfermedades 10 (CIE-10), para permitir una mejor visualización. Las principales causas registradas fueron enfermedades del sistema circulatorio (n=1.593, 28,6%), neoplasias (n=893, 16%), enfermedades del sistema genitourinario (n=508, 9,1%), enfermedades del sistema digestivo (n=504, 9%) y enfermedades del sistema respiratorio (n=446, 8%).

Cuando se extrajeron los valores para identificar los atributos relacionados con el dolor se identificaron 44.093 cuidados de enfermería prescritos para el dolor, distribuidos en cinco principales diagnósticos de enfermería (DE) con cuidados prescritos para el dolor, a saber: Dolor Agudo (73,7%), Dolor Crónico (9,5%), Riesgo de Caídas (8,9%), Deterioro del Confort (4,8%) y Deterioro de la Movilidad Física (1,7%), además de otros diagnósticos de enfermería en los que también se habían prescrito cuidados para el dolor con un porcentaje menor (1,4%).

Las principales etiologías relacionadas con el dolor enumeradas en el DE fueron las siguientes: trauma relacionado con un procedimiento invasivo y/o lesión quirúrgica (57,3%); progresión de la enfermedad (20%); deterioro de la movilidad (7,6%); problema vascular (4,4%); agentes biológicos, químicos, físicos y psicológicos perjudiciales (7,6%); síntomas de enfermedad o dolor (4,2%); deterioro de la movilidad física (3,6%). Cabe destacar que el valor “dolor” estuvo presente como DE y también como etiología de un determinado diagnóstico, por ejemplo, deterioro de la movilidad física. Sin embargo, para la extracción de datos se consideraron los atributos prescritos en los cuidados de enfermería, con el fin de evaluar en qué DE había prescripción de cuidados para el dolor con mayor frecuencia para la población de personas mayores de 75 años o más.

Cuando las etiologías se estratificaron por DE se observó que las distribuciones eran muy diferentes entre ellas. Las principales etiologías del DE Dolor Agudo fueron trauma relacionado con un procedimiento invasivo y/o lesión quirúrgica, progresión de la enfermedad y problema vascular. Las principales etiologías del DE Dolor Crónico registradas fueron evolución de la enfermedad y proceso inflamatorio. Cuando se prescribieron cuidados para el dolor en el DE Riesgo de Caídas, la principal etiología fue deterioro de la movilidad.

 Se identificaron 60 atributos relacionados con el dolor en los adultos mayores vulnerables en diferentes tablas de bases de datos, en la Figura 2 se describen los principales atributos en función del número de observaciones. Los atributos identificados se organizaron en siete grupos: (1) Dolor actual, (2) Instrumentos y características de evaluación del dolor, (3) Título de los Diagnósticos de Enfermería, (4) Etiología de los Diagnósticos de Enfermería, (5) intervenciones para aliviar el dolor, (6) Consultoría de especialidad y (7) Reevaluación del dolor. La organización en grupos sirvió de base para elaborar las definiciones conceptuales de los grupos de atributos y mejorar la comprensión semántica del MI desarrollado. 

**Figura 2 -:** Principales atributos encontrados sobre el manejo del dolor en los adultos mayores vulnerables en los registros médicos electrónicos

Los siete grupos diferentes de atributos se agruparon y organizaron en paneles de clasificación: (1) Panel de Evaluación; (2) Panel de Intervenciones; (3) Panel de Diagnósticos de Enfermería y sus componentes; y (4) Panel de Reevaluación.

 La clasificación de los atributos se basó en un MI de manejo del dolor validado ^(^ 2018 ^)^ , que incorporó en su elaboración aspectos relacionados a la cultura institucional y al diagrama de flujo de la organización del sistema de información hospitalario. Por lo tanto, a partir de los RES, se organizaron los componentes relacionados con el manejo del dolor en los adultos mayores vulnerables. En la Figura 3 se describen la definición conceptual de la clasificación y los grupos de atributos para comprender la semántica del MI desarrollado. 

 Por último, a partir de los datos de RES relacionados con el dolor que se extrajeron en la muestra del estudio y después de clasificar y organizar estos atributos, se desarrolló un MI para el manejo del dolor en los adultos mayores vulnerables en un hospital universitario del sur de Brasil. El MI desarrollado se representa en una estructura visual en la Figura 4 . 

**Figura 3 -:** Definición descriptiva de los grupos de atributos y clasificación (paneles) utilizados en el desarrollo del Modelo de Información

## Discusión

A partir de los RES estructurados de los adultos mayores de 75 años o más se desarrolló el primer MI sobre el manejo del dolor en este grupo poblacional vulnerable. Aunque la base de datos se restringe solamente a datos estructurados, la extracción mediante técnicas de ciencia de datos permitió obtener información extensa y con un volumen importante de la muestra en estudio.

 Ya se desarrollaron previamente MI sobre el manejo del dolor con grandes bases de datos en Estados Unidos ^(^ 2017 ^)^ , y también en Brasil ^(^ 2021 ^)^ , pero no específicamente para los adultos mayores. El MI estadounidense sobre manejo del dolor ^(^ 2017 ^)^ , con datos de varias instituciones de salud, fue validado por la evaluación de expertos ^(^ 2018 ^)^ , lo que mejoró su aplicabilidad clínica y brinda perspectivas para la interoperabilidad entre sistemas de salud. El MI desarrollado en el presente estudio se desarrolló con datos de una sola institución hospitalaria, lo que reduce su generalización. 

 La estratificación por edad entre pacientes adultos mayores y los adultos mayores vulnerables, de 75 años o más, es una herramienta para operacionalizar el seguimiento de los adultos mayores hospitalizados en la institución ^(^ 2019 ^)^ . Sin embargo, la relevancia de la edad en la prevalencia del dolor es controvertida en la literatura ^(^ 2018 ^)^ . No se observó que la edad fuera un factor significativamente diferente entre las personas mayores con y sin dolor corporal ^(^ 2021 ^)^ , en el dolor de rodilla ^(^ 2020 ^-^ 2023 ^)^ . Sin embargo, la edad es un factor significativo en la intensidad del dolor en pacientes con lumbalgia ^(^ 2023 ^)^ , después de una artroplastia total de cadera ^(^ 2022 ^)^ y en los signos de cambio en la percepción del dolor después de un accidente cerebrovascular ^(^ 2022 ^)^ . 

 Dichos escenarios demuestran la complejidad del dolor en los adultos mayores y que es necesario desarrollar MI que evalúen los cuidados relacionados con el manejo del dolor en grupos específicos. El MI desarrollado en este estudio permitió clasificar los atributos extraídos del dolor en evaluación, intervenciones y reevaluación del dolor. Estos cuatro paneles principales de clasificación del manejo del dolor en la institución se originaron a partir de los siete grupos de atributos relacionados con el dolor, basados ​​en el MI estadounidense validado ^(^ 2017 ^)^ . 

 Se observa en los RES que se utilizan instrumentos unidimensionales en la evaluación del dolor ^(^ 2019 ^)^ que sólo permiten evaluar la intensidad, tales como las escalas analógica visual, numérica verbal y categórica verbal. En general, las escalas unidimensionales son buenos instrumentos para evaluar el dolor y de fácil uso en la práctica clínica ^(^ 2018 ^-^ 2018 ^)^ , pero para los adultos mayores estas herramientas pueden no ser suficientes, debido a las discapacidades cognitivas, las dificultades para comunicar la experiencia dolorosa y los factores conductuales de dicho público ^(^ 2018 ^,^ 2019 ^)^ . 

**Figura 4 -:** Modelo de Información para el manejo del dolor en los adultos mayores con 75 años o más

 Los instrumentos multidimensionales para evaluar el dolor permiten conocer mejor los aspectos psicológicos, sociales y económicos que intervienen en la experiencia dolorosa y también realizar una anamnesis más sólida para los adultos mayores ^(^ 2019 ^)^ . Existen cinco instrumentos multidimensionales para evaluar el dolor validados en Brasil ^(^ 2016 ^-^ 2006 ^)^ , algunos de los cuales están adaptados para ancianos con limitada capacidad de comunicación o para pacientes no comunicativos ^(^ 2016 ^-^ 2012 ^)^ , además de instrumentos específicos para los adultos mayores con demencia ^(^ 2016 ^)^ . En la institución del estudio no se utiliza ninguno de esos instrumentos, lo que constituye una debilidad en la atención integral de la salud de los adultos mayores. 

 En los RES se observó mayor variabilidad en los cuidados relacionados con el dolor prescritos por los enfermeros, que abarcan aspectos fisiológicos, psicológicos y sociales involucrados en el manejo del dolor. Sin embargo, el volumen de las observaciones indica que se le da mayor importancia a la evaluación y a las intervenciones farmacológicas para el dolor, resultado similar al que arrojó otro estudio brasileño ^(^ 2020 ^)^ . Los MI contribuyen en este sentido, porque permiten comprender las relaciones y complejidades del flujo de información sobre temas de interés para la práctica de enfermería ^(^ 2021 ^,^ 2017 ^)^ . 

En la elaboración del MI se decidió incluir como uno de los grupos representados el DE y sus componentes, debido a la gran cantidad de registros estructurados, que representan el valor institucional que tienen para la aplicación del proceso de enfermería. En el MI desarrollado se incluyeron tres DE con cuidados prescritos para el dolor, además de aquellos que son específicos (DE Dolor Agudo y DE Dolor Crónico), a saber: Riesgo de Caídas, Deterioro del Confort y Deterioro de la Movilidad Física.

 Estos tres DE se refieren al deterioro de la funcionalidad motora y de la movilidad física, eventos comunes en adultos mayores, especialmente en los que se encuentran hospitalizados ^(^ 2021 ^-^ 2021 ^)^ . Es importante resaltar que los enfermeros diagnostican problemas de salud y condiciones de riesgo sobre una respuesta humana que puede o no estar asociada a otras condiciones médicas ^(^ 2021 ^)^ . Por lo tanto, el deterioro de la movilidad que lleva al enfermero a enumerar los DE Riesgo de Caídas, Deterioro del Confort o Deterioro de la Movilidad Física identifican ese signo como una necesidad de cuidado en ese momento. Puesto que las respuestas humanas son dinámicas, los DE también lo son, y reflejan una necesidad en el momento en que se evaluó y esta demanda puede haber comenzado antes o durante la hospitalización. 

 Dado que, para elaborar nuestro MI para el manejo del dolor, extraemos valores presentes en la atención prescrita, los datos encontrados en este análisis sugieren que los signos y síntomas de dolor pueden estar asociados con un mayor riesgo de caídas en los adultos mayores vulnerables, considerando la frecuencia de los cuidados prescriptos para el dolor en el DE Riesgo de Caídas, el tercer DE con el mayor volumen de cuidados prescriptos para el dolor. Esta característica no está presente en los MI brasileños sobre manejo del dolor desarrollados con pacientes adultos ^(^ 2021 ^)^ . 

 Diferentes estudios ^(^ 2021 ^-^ 2021 ^)^ indican que no se observó que el riesgo de caídas fuera una variable asociada al dolor, independientemente de su intensidad, o un factor protector contra la aparición de caídas ^(^ 2020 ^)^ . Por otro lado, un estudio realizado con adultos de mediana edad y adultos mayores identificó que la presencia de signos de dolor era un factor que aumenta el riesgo de caídas ^(^ 2020 ^)^ , al igual que un estudio ^(^ 2021 ^)^ realizado con los adultos mayores con 70 años o más. Debido a las características descriptivas del MI desarrollado, no se puede afirmar si hay asociación entre el dolor y el riesgo de caídas. Es necesario realizar análisis más detallados sobre esta relación, dado que es una laguna que se debe estudiar más a fondo. 

 Existe fuerte evidencia de que una menor red de apoyo para los adultos mayores se relaciona tanto con el riesgo de caídas como con un peor manejo del dolor ^(^ 2022 ^)^ , además es un factor relacionado a la capacidad funcional en esta población ^(^ 2020 ^)^ . Esto, por sí solo, indica que es necesario que se implementen protocolos para el traje y seguimiento de estos pacientes que deben ser evaluados de forma multidimensional, con el objetivo de organizar la red de apoyo en la planificación del alta. 

Este estudio tuvo limitaciones en su diseño que deben ser consideradas al evaluar los resultados presentados. El desarrollo del MI sólo se basó en datos estructurados de las historias clínicas electrónicas de los pacientes, no incluyó documentos considerados importantes como la evolución y anamnesis y el examen físico de enfermería, porque son registros de texto libre.

Al tratarse de un estudio que utiliza registros secundarios, también está sujeto a sesgos de información, como errores de registro o datos faltantes, como la fecha de hospitalización de los pacientes, que se utilizó para determinar posteriormente la edad de la muestra. Para abordar esa limitación, dicha variable fue tratada de manera que se considerara como fecha de hospitalización del paciente la primera aparición de una prescripción médica o de enfermería registrada.

Otra limitación importante fue el mapeo mediante análisis exploratorio y la creación de los comandos de programación por parte de un solo investigador, lo que está sujeto a errores al extraer la información de la base de datos. Además, los atributos sobre el manejo del dolor en los adultos mayores vulnerables se mapearon en una sola institución y en las historias clínicas de los pacientes ingresados en unidades clínicas y quirúrgicas.

 En cuanto a los aspectos de gestión e investigación en salud, los MI son la parte inicial de la creación de modelos semánticos en los que se basan los proyectos de interfaz de sistemas de información en salud. Tanto el MI desarrollado en este estudio como el MI para el manejo del dolor en adultos desarrollado en Brasil ^(^ 2021 ^)^ fueron realizados a partir de los datos de una sola institución. En futuras propuestas, pueden servir de base para la interoperabilidad de datos entre más de una institución, pueden contribuir a construir una atención orientada por datos y con mucho mayor potencial de generalización. 

## Conclusión

El MI nos permitió obtener una visión general de la realidad asistencial del manejo del dolor en los adultos mayores hospitalizadas de 75 años o más. El desarrollo del MI permitió organizar los registros relacionados con el manejo del dolor, clasificándolos en grandes grupos que reflejan la atención brindada a los adultos mayores, así como los recursos institucionales utilizados para el manejo del dolor en esta población.

Se observa madurez institucional en cuanto a la aplicación del proceso de enfermería de manera estructurada, además de la presencia de diferentes tipos de intervenciones de enfermería que incluyen aspectos biológicos, psicológicos y psicosociales del cuidado de enfermería.

Con respecto a las lagunas asistenciales observadas, se identificó que no había instrumentos multidimensionales para el manejo del dolor en los registros, lo que dificulta identificar ese signo en los adultos mayores con deterioro funcional o déficits en la comprensión y comunicación del dolor a través de escalas numéricas.

El estudio permitió observar que el deterioro de la movilidad física aparece como un factor prevalente en los registros de los cuidados prescritos para el dolor a los adultos mayores vulnerables. Se deduce que la presencia de dolor junto con el deterioro de la movilidad en los adultos mayores puede aumentar el riesgo de episodios de caídas. Sin embargo, sería necesario realizar estudios con diseños más robustos que permitan evaluar esta asociación.

El MI desarrollado no fue validado. En un escenario prospectivo, la validación e implementación de MI puede servir como base para la interoperabilidad semántica entre sistemas de información o para el desarrollo de modelos predictivos para el manejo del dolor, especialmente para la población de ancianos; además de optimizar los sistemas de información hospitalarios.

# Translation: Modelo de informação do gerenciamento da dor em pessoas idosas com 75 anos ou mais*

**DOI:** 10.1590/1518-8345.7111.4306

**Published:** 2024-10-09

**Authors:** Ana Clara de Brito Cruz, Amália de Fátima Lucena, Aline Tsuma Gaedke Nomura, Miriam de Abreu Almeida

**Affiliations:** ^1^ Universidade Federal do Rio Grande do Sul, Escola de Enfermagem, Porto Alegre, RS, Brasil; ^2^ Bolsista da Fundação de Amparo à Pesquisa do Estado do Rio Grande do Sul (FAPERGS), Brasil; ^3^ Bolsista do Conselho Nacional de Desenvolvimento Científico e Tecnológico (CNPq), Brasil; ^4^ Bolsista da Coordenação de Aperfeiçoamento de Pessoal de Nível Superior (CAPES), Brasil; ^5^ Hospital de Clínicas de Porto Alegre, Serviço de Enfermagem Cardiovascular, Nefrologia e Imagem, Porto Alegre, RS, Brasil

**Keywords:** Manejo da Dor, Saúde do Idoso, Cuidado de Enfermagem ao Idoso Hospitalizado, Registros Eletrônicos de Saúde, Informática em Enfermagem, Mineração de Dados

## Abstract

desenvolver um modelo de informação sobre o gerenciamento da dor em pessoas idosas hospitalizadas.

estudo observacional retrospectivo de *Big Data,* guiado pelo *Applied Healthcare Data Science Roadmap* . A amostra incluiu todos os Registros Eletrônicos de Saúde relacionados ao gerenciamento da dor em pessoas idosas com 75 anos ou mais, considerados vulneráveis na instituição, internados em unidades clínicas e cirúrgicas. Para análise foram utilizados pacotes de ciência de dados em Python ^®^

encontrou-se 9.635 internações referentes a 4.753 pacientes, com média de 81 anos, sendo 54% do sexo feminino. Os principais motivos de hospitalização foram doenças do aparelho circulatório (n = 1.593, 28,6%), neoplasias (n = 893, 16%) e doenças do aparelho geniturinário (n = 508, 9,1%). Identificou-se 60 atributos relacionados à dor, organizados nos grupos: dor atual; instrumentos e características de avaliação; diagnóstico de enfermagem; etiologia do diagnóstico de enfermagem; intervenções para alívio; consultorias com especialidades; e reavaliação da dor. Os grupos foram classificados em quatro grandes painéis que constituíram o modelo de informação

o modelo de informação desenvolvido apresentou um panorama da realidade assistencial do gerenciamento da dor em pessoas idosas vulneráveis, subsidiando a tomada de decisão para a gestão da dor nesta população

## Destaques

**(1)** Classificou-se os atributos em: avaliação, intervenções e reavaliação da dor.

**(2)** Observa-se maior importância dada à avaliação e às intervenções farmacológicas da dor.

**(3)** Houve uma variabilidade maior nos cuidados para dor prescritos por enfermeiros.

**(4)** Identificou-se a ausência de instrumentos multidimensionais para avaliação da dor.

**(5)** Cuidados para dor foram prescritos de forma recorrente em idosos com risco de quedas.

## Introdução

 Tende-se a esperar que a dor aumente com a idade, seja devido ao acúmulo de lesões musculoesqueléticas, desgaste físico com o envelhecimento ou pela presença de multimorbidades ^(^ 2022 ^-^ 2020 ^)^ . Entretanto, a dor na pessoa idosa é complexa, pois é associada a fatores psicossociais em conjunto às alterações da modulação deste sinal, que ainda não são bem esclarecidos ^(^ 2022 ^)^ . A avaliação clínica habitual pode não englobar essas particularidades da pessoa idosa, especialmente no contexto hospitalar ^(^ 2019 ^)^ . 

 Diferentes estratégias foram desenvolvidas por programas hospitalares para suprir as necessidades integrais da pessoa idosa hospitalizada, principalmente a prevenção de declínio funcional ^(^ 2022 ^)^ . A idade é um fator determinante no desenvolvimento de incapacidades durante a hospitalização, visto que pacientes a partir dos 75 anos apresentam maior probabilidades de perder a capacidade de executar atividades de vida diária durante a hospitalização ^(^ 2003 ^)^ . Com uma avaliação geriátrica abrangente, pessoas idosas têm melhores chances de sobreviver à hospitalização sem sofrer deterioração cognitiva ou funcional ^(^ 2022 ^)^ . 

 Partindo deste prisma, pesquisadores de um hospital universitário de grande porte, localizado no sul do Brasil, desenvolveram um protocolo de cuidado a pessoas idosas hospitalizadas ^(^ 2019 ^)^ . Considerando o envelhecimento populacional e a operacionalidade do serviço hospitalar, há um foco direcionado a pessoas idosas mais velhas e que estão mais vulneráveis ao risco de sofrerem deterioração funcional durante a hospitalização ^(^ 2003 ^)^ . O protocolo institucional ^(^ 2019 ^)^ , portanto, considera todas as pessoas a partir de 75 anos como vulneráveis. 

 Nesse contexto, a dor é um sinal também relacionado à redução da capacidade funcional e da piora da qualidade de vida ^(^ 2022 ^-^ 2020 ^,^ 2003 ^)^ , constituindo-se um aspecto importante a ser considerado na elaboração do plano de cuidados à pessoa idosa hospitalizada. 

 Para o registro do plano de cuidados, as instituições hospitalares aplicam de forma cada vez mais usual os sistemas de informação eletrônicos, que acabam servindo como uma fonte de informações promissora. Registros secundários coletados na prática assistencial, ou Registros Eletrônicos de Saúde (RES), contêm um grande volume de informações oriundas dos serviços de saúde e produzidas continuamente por diferentes profissionais ^(^ 2021 ^)^ . Assim, uma das potencialidades do uso de RES é a análise de informações de saúde vinculadas às ferramentas da ciência de dados, como análises de *Big Data* ^(^ 2021 ^-^ 2020 ^)^ . 

 Em suma, o termo *Big Data* refere-se às características dos banco de dados utilizado, como grande volume de dados, acúmulo rápido de novas informações e variedade de dados, devido aos formatos diferenciados ^(^ 2020 ^)^ . Os métodos de análise de *Big Data* referem-se a técnicas e métodos de análise de uma grande quantidade de RES. Esses dados são muito diversos e difíceis de visualizar através de *softwares* ou ferramentas tradicionais em pesquisa, demandando uso de conhecimentos e ferramentas específicos para processá-los ^(^ 2021 ^-^ 2020 ^)^ . 

 Logo, as análises de *Big Data* podem ser utilizadas por enfermeiros para aprimorar processos de trabalho sensíveis ao cuidado de enfermagem, tanto no cenário assistencial, quanto na gestão em saúde ^(^ 2021 ^-^ 2021 ^)^ . Tais abordagens analíticas permitem o desenvolvimento da “enfermagem de precisão”, visando oferecer um cuidado personalizado, adequado às particularidades de cada indivíduo, caminhando em direção a modelos de saúde preditivos ^(^ 2021 ^,^ 2020 ^)^ . Uma das estratégias utilizadas em instituições de saúde norte-americanas para adequar a organização de RES, aproveitando a potencialidade do uso desses registros, é a elaboração de Modelos de Informação (MI) ^(^ 2017 ^)^ . 

 Os MI elucidam, de forma visual, conceitos e atributos presentes nos RES, permitindo que as informações sejam visualizadas e acessadas de forma mais eficiente, tornando os dados mais compreensíveis e facilitando a utilização das informações coletadas para decisões clínicas e gerenciais ^(^ 2021 ^,^ 2017 ^)^ . Além disso, também oferecem suporte para construção de ontologias ^(^ 2017 ^-^ 2019 ^)^ , que são um modelo formal de conceitos, propriedades, relações e funções entre informações de um banco de dados. Um MI de alto nível, com conceitos e relações bem definidos, permite a interoperabilidade de dados clínicos entre diferentes sistemas de informação e instituições de saúde ^(^ 2021 ^,^ 2017 ^)^ . 

 Diferentes MI relacionados à dor e a outros temas sensíveis à prática de enfermagem já foram produzidos, principalmente com dados secundários de hospitais estadunidenses ^(^ 2017 ^)^ . No contexto brasileiro, foi desenvolvido um MI do gerenciamento da dor utilizando dados de RES de pacientes adultos ^(^ 2021 ^)^ , que contou com mais de 50 mil pacientes. Entretanto, a ampla faixa etária abrangida no estudo - dos 18 aos 107 anos - dificulta a identificação de necessidades de cuidado e particularidades relacionadas à dor em populações específicas, como a população de pessoas idosas. 

 Tendo em vista que a dor é um sinal que tende a estar mais presente na população acima dos 70 anos ^(^ 2020 ^)^ e é impactante sobre a capacidade funcional ^(^ 2022 ^-^ 2020 ^)^ , identificou-se a necessidade de aprimorar o conhecimento relacionado ao gerenciamento da dor em pessoas idosas. A construção de um MI do gerenciamento da dor em pessoas idosas vulneráveis, a partir dos 75 anos, oferece diversos benefícios potenciais que vão ao encontro da qualificação e segurança do paciente; assim como do aprimoramento do cuidado de enfermagem à pessoa idosa hospitalizada. 

Dessa forma, o presente estudo tem por objetivo desenvolver um modelo de informação sobre o gerenciamento da dor em pessoas idosas hospitalizadas, buscando responder à questão norteadora: “Como o gerenciamento da dor em pessoas idosas vulneráveis pode ser observado em RES?”.

## Método

### Delineamento do estudo

 Trata-se de um estudo observacional retrospectivo, com utilização de dados secundários, utilizando o processo de *data-driven* para análise dos RES. *Data-driven* é um processo organizacional no qual se faz uso do mapeamento da informação para direcionar o planejamento e o processo de tomada de decisões ^(^ 2017 ^)^ . 

### Local do estudo

 O estudo foi realizado a partir de registros eletrônicos em saúde provenientes do Hospital de Clínicas de Porto Alegre (HCPA), que é geral e público, vinculado à Universidade Federal do Rio Grande do Sul, o que o caracteriza como hospital universitário ^(^ 2023a ^)^ . No que tange à gestão hospitalar e à assistência em saúde, o HCPA é apoiado pelo uso da tecnologia da informação, principalmente, representada pelo Aplicativo para Gestão Hospitalar denominado AGHUse. Esse sistema facilita o gerenciamento e o registro das atividades assistenciais e administrativas em saúde e foi desenvolvido pela própria instituição, sendo atualmente compartilhado com outras seis instituições públicas no país ^(^ 2023b ^)^ . 

 No HCPA, o envelhecimento é entendido como um indicador que determina necessidades específicas de assistência baseado em um Protocolo Assistencial ao Idoso Hospitalizado ^(^ 2019 ^)^ . Todas as pessoas idosas, a partir de 60 anos, são avaliadas quanto ao risco de vulnerabilidade pelo enfermeiro por meio da escala de avaliação PRISMA-7 ^(^ 2008 ^)^ . A partir dos 75 anos, todas as pessoas idosas são consideradas vulneráveis, ou seja, estão mais suscetíveis a riscos de perda de funcionalidade mental e funcional decorrente da hospitalização e são avaliados de forma multiprofissional ^(^ 2019 ^)^ . 

 Apesar do instrumento PRISMA-7 ^(^ 2008 ^)^ ter sido desenvolvido voltado para idosos com 85 anos ou mais, o HCPA utiliza-o de forma adaptada para rastreamento de pessoas idosas que venham a necessitar de acompanhamento multiprofissional de forma periódica, considerando que é um instrumento de aplicação rápida e de fácil compreensão para as equipes assistenciais que realizam o rastreamento. 

 As pessoas idosas, a partir de 75 anos, são acompanhadas pela equipe multiprofissional, enquanto as pessoas idosas mais jovens são acompanhadas de acordo com a identificação de maior vulnerabilidade, conforme o instrumento PRISMA-7. Trata-se de uma escolha gerencial, feita pela instituição, como uma forma de otimizar os acompanhamentos na linha de cuidado à pessoa idosa hospitalizada, visto que há uma crescente pela demanda dos serviços da instituição por pessoas cada vez mais velhas, como foi observado no ano de 2023 com aumento de 40% no número de internações de pessoas com 75 anos ou mais em comparação com o ano anterior ^(^ 2024 ^)^ . 

 Quanto ao Processo de Enfermagem na instituição, este é informatizado e tem como referencial teórico de enfermagem as Necessidades Humanas Básicas ^(^ 2016 ^)^ , além disso, o sistema eletrônico contempla todas as etapas do processo de enfermagem. Na etapa de diagnóstico de enfermagem (DE) é utilizada a terminologia da NANDA-Internacional (NANDA-I) ^(^ 2021 ^)^ , com adaptações para a estrutura do sistema eletrônico e para a realidade institucional. Dentro das adaptações feitas, notam-se os itens expressos na terminologia da NANDA-I, como: fatores relacionados, características definidoras, fatores de risco e populações em risco, que estão compilados no sistema eletrônico com o termo “etiologia”. 

 Já as intervenções de enfermagem estão associadas aos diagnósticos de enfermagem e são baseadas na terminologia da Classificação das Intervenções de Enfermagem - *Nursing Interventions Classification* (NIC) ^(^ 2020 ^)^ e na literatura relacionada à prática clínica. Por sua vez, no AGHUse as intervenções de enfermagem são referidas pelo termo “cuidados”. 

### Período

Os dados do estudo são referentes ao período entre julho de 2014 e junho de 2019, ao passo que o desenvolvimento do MI do gerenciamento da dor em pessoas idosas vulneráveis se deu no período entre fevereiro de 2022 e março de 2023.

### População e amostra

 A população do estudo foi composta por todos os RES estruturados, originários dos prontuários eletrônicos, com dados clínicos e sociodemográficos de pacientes adultos internados em unidades clínicas e cirúrgicas do HCPA. A população representa os RES coletados em projeto de pesquisa anterior com pacientes adultos ^(^ 2021 ^)^ . A amostra compreendeu o recorte de registros referente às pessoas idosas a partir de 75 anos; contabilizando mais de três milhões de observações em RES pertencentes a 7.453 pacientes. 

### Coleta de dados

Para elaboração deste estudo, utilizou-se dados clínicos e sociodemográficos estruturados, registrados em prontuário eletrônico, relacionados ao gerenciamento da dor em pessoas idosas com 75 anos ou mais e considerados pela instituição como mais vulneráveis.

 E, ainda, este estudo utilizou-se de *queries* para seleção dos RES no banco de dados do hospital. As *queries* foram construídas pelos analistas de tecnologia da informação da instituição hospitalar na qual a pesquisa se desenvolveu, e foram compartilhadas com os pesquisadores envolvidos através de tabelas em *Excel.* O banco de dados bruto em *Excel* , referente ao projeto de pesquisa maior, foi transformado em CSV ( *Comma-Separated Values* ) com o objetivo de permitir a utilização de técnicas de manuseio de *Big Data* , considerando o volume de dados. 

 Os critérios para construção das *queries* foram: todos os registros eletrônicos estruturados de pacientes adultos (acima de 18 anos), internados em unidades clínicas e cirúrgicas. A partir desse banco de dados delineou-se um recorte para o aprofundamento da investigação da dor na população idosa com 75 anos ou mais, o que compõe o presente estudo. 

 O manuseio dos RES foi guiado pelo roteiro de ciência de dados denominado *Applied Healthcare Data Science Roadmap* ^(^ 2020 ^)^ . Esta metodologia engloba seis etapas: (1) Entender a questão de pesquisa; (2) Análise exploratória dos dados; (3) Preparação dos dados; (4) Modelagem Analítica; (5) Avaliação ou validação; (6) Implementação ou desdobramento. Em vista disso, este estudo engloba as quatro primeiras etapas do roteiro. 

Além do mais, nesta metodologia, as variáveis são obtidas por meio da exploração do banco de dados retrospectivo em um ambiente de programação, o que permite o rastreio de todos os pacientes. Dessa forma, avaliamos os pacientes com 75 anos ou mais encontrados no banco de dados.

 Neste estudo são considerados apenas os dados estruturados, ou seja, que são organizados no formato de tabelas. As tabelas que compuseram o banco de dados de pessoas idosas com 75 anos ou mais e o volume de informações obtidas pode ser observado na Tabela 1 . 

Cada coluna das tabelas representa uma variável a ser explorada, enquanto cada linha representa uma observação das variáveis presentes nas tabelas. O número de pacientes com 75 anos ou mais varia de tabela para tabela, considerando que algumas intervenções – por exemplo, solicitação de consultoria a especialidades – não foram solicitadas para todos os pacientes. Dessa forma, para construção do MI, os autores consideraram as observações totais de cuidados prescritos para dor, e não o número de observações por paciente.

 A representação da coleta de dados na base do AGHUse, as tabelas e as variáveis utilizadas no banco de dados dos pacientes com 75 anos ou mais estão representados na Figura 1 . 

**Tabela 1 -:** Tabelas que compuseram o banco de dados de pessoas idosas com 75 anos ou mais. Porto Alegre, RS, Brasil, 2019

**Título da tabela**	**Nº de pacientes únicos**	**Nº de variáveis (colunas)**	**Nº de observações (linhas)**
Consultoria às especialidades	2573	5	13978
Medicamentos prescritos	3230	10	342463
Cuidados médicos	3291	8	341531
Nutrição parenteral	44	5	1142
Comorbidades prévias	3294	3	19943
Procedimentos médicos	1596	10	31644
Motivo da internação	1453	4	5545
Dieta	3294	10	88694
Soluções	2915	9	81249
Cuidados de enfermagem	3270	15	769420
Hemoterapia	2588	6	6624
Sinais vitais	2924	6	1840097
Sociodemográficos	4748	6	4748

* CID = Código Internacional das Doenças - CID10

**Figura 1:** - Representação da coleta e preparação do banco de dados que compuseram os registros eletrônicos de saúde das pessoas idosas vulneráveis

### Tratamento e análise dos dados

No tratamento e análise foram realizadas as etapas de análise exploratória; bem como a preparação e a modelagem dos dados relacionados à dor em pacientes com 75 anos ou mais. A análise exploratória refere-se à observação ampla dos dados disponíveis e rastreamento de pacientes com 75 anos ou mais. Na preparação dos dados são realizadas as adequações do banco de dados para rastreamento dos atributos relacionados ao gerenciamento da dor; na modelagem analítica é realizada a categorização dos atributos encontrados.

### Análise exploratória dos dados

 Foi organizado um *Integrated Development Environment* (IDE) para manuseio do banco de dados no Jupyter *notebook* em linguagem de programação Python ^®^ e utilizado pacotes da biblioteca Pandas para análise de dados ^(^ 2018 ^)^ . 

 No IDE foram explorados os conteúdos de cada variável presente nas tabelas que compuseram o banco de dados, a fim de identificar as informações relacionadas ao gerenciamento da dor em pessoas idosas. Cada paciente único possuía um identificador numérico, aqui denominado de “identificador único do paciente”, que foi utilizado como identificador-chave ^(^ 2018 ^)^ , com o propósito de cruzar as diferentes tabelas e garantir que os dados fossem referentes aos pacientes com 75 anos ou mais. 

### Preparação dos dados

No pré-processamento foram realizadas as adequações necessárias no banco de dados pela exclusão de dados que possibilitassem a identificação do paciente ou de profissionais envolvidos no cuidado, assim como a exclusão de dados incoerentes (por exemplo: erros de digitação, como o uso de caracteres ao invés de números).

Para determinar a idade dos pacientes, foi considerado o dado estruturado de data de nascimento associado à data da primeira internação; uma vez que um único paciente pode ter apresentado mais de uma internação no período estudado. Pacientes sem data de nascimento e/ou sem data de internação foram excluídos.

Além disso, para minerar os dados relacionados à dor que estavam disponíveis no banco de dados, foram buscados os termos “analgesia”, “doloros*”, “dor” e “antálgic*” em todas as tabelas, exceto nos registros de medicações prescritas. Neste caso, as medicações relacionadas à dor foram mineradas por códigos de identificação da instituição, que permitiu identificá-los entre analgésicos opioides e não opioides. As informações mineradas foram contabilizadas por número de observações e foi realizado o cálculo de distribuição dos dados. Também foram utilizados como fontes de dados para desenvolvimento do MI os dados de anotações de enfermagem (sinais vitais e níveis de dor); diagnósticos e prescrições de enfermagem; assistência médica não farmacológica; diagnósticos médicos; medicamentos prescritos; procedimentos médicos; além de dados sociodemográficos.

 Todavia, registros com menos de 50 observações foram considerados insuficientes e desconsiderados para elaboração do MI, conforme estudo prévio ^(^ 2021 ^)^ . Os atributos relacionados ao gerenciamento da dor em pessoas idosas foram agrupados e categorizados. 

### Modelagem analítica

 Após a preparação do banco de dados, as informações obtidas relacionadas ao gerenciamento da dor na população de estudo foram categorizadas. A categorização foi baseada em um MI de gerenciamento da dor ^(^ 2018 ^)^ norte-americano validado entre 10 instituições hospitalares, e leva em consideração as características culturais institucionais do local de estudo e o processo de enfermagem adotado na instituição. 

 A partir da categorização, os conceitos e os atributos foram desenhados, com uma estrutura visual de relações entre os resultados obtidos, utilizando o *software* *LucidChart* . A estrutura visual desenvolvida trata-se do MI do gerenciamento da dor em pessoas idosas a partir de 75 anos. Esta modelagem observacional permite a compreensão abrangente dos processos e dos atributos presentes nos RES e que retratam as práticas de cuidado realizadas na instituição. 

### Aspectos éticos

Para manutenção do sigilo e da segurança da informação, o número de prontuário eletrônico de todos os pacientes foi de-identificado pela instituição hospitalar, sendo fornecida às pesquisadoras uma identificação numérica única por paciente nas tabelas. O projeto de pesquisa foi cadastrado na Plataforma Brasil, encaminhado ao Comitê de Ética em Pesquisa do Hospital de Clínicas de Porto Alegre e aprovado sob registro nº 2018.0669.

## Resultados

 Para o desenvolvimento do MI sobre o gerenciamento da dor, foram utilizadas metodologias específicas para a manipulação de *Big Data* . Ainda assim, durante a análise exploratória foram necessários a limpeza e o tratamento dos dados para extrair somente as informações pertinentes ao objetivo do estudo. 

Identificou-se 4.753 pacientes, referentes a 9.635 internações no período estudado. A média de idade dos pacientes internados foi de 81 anos, sendo o mais velho de 107 anos. Quando estratificados por faixas etárias, 46,6% deles estavam entre os 75 e 79 anos; 30,1% entre os 80 e 84 anos; 16,7% entre 85 e 89 anos; e 6,6% com 90 anos ou mais. Dentre os 4.753 pacientes, 2.579 (54,2%) eram do sexo feminino, 4.413 (92,8%) de pessoas brancas. Outros grupos raciais foram encontrados em menores proporções: 250 pessoas pretas (5,2%), 82 pardas (1,7%) e oito amarelas (0,15%).

Quanto ao nível de instrução, a maioria dos pacientes possuía educação básica incompleta (n = 2.215, 46,7%), seguido pela educação básica completa (n = 854, 17,9%), ensino médio completo (n = 600, 12,7%), ensino superior completo (n = 396, 8,3%), nenhuma instrução formal (n = 216, 4,5%), ensino médio incompleto (n = 100, 2,1%) e ensino superior incompleto (n = 36, 0,7%). Houve ausência desse registro em 336 (7,1%) casos.

Ademais, o motivo da internação em registro estruturado foi encontrado em 5.562 internações referentes a 1.457 pacientes. Desse total de internações, as causas foram agrupadas em grandes grupos do Código Internacional das Doenças - CID10, para permitir melhor visualização. As principais causas registradas foram as doenças do aparelho circulatório (n = 1.593, 28,6%), neoplasias (n = 893, 16%), doenças do aparelho geniturinário (n = 508, 9,1%), doenças do aparelho digestivo (n = 504, 9%) e as doenças do aparelho respiratório (n = 446, 8%).

Quando minerados os valores para identificação dos atributos relacionados à dor, foram identificados 44.093 cuidados de enfermagem prescritos para dor, distribuídos entre cinco principais diagnósticos de enfermagem (DE) com cuidados prescritos para dor, sendo eles: Dor Aguda (73,7%), Dor Crônica (9,5%), Risco de Quedas (8,9%), Conforto Prejudicado (4,8%) e Mobilidade Física Prejudicada (1,7%), além de outros diagnósticos de enfermagem que também possuíam cuidados prescritos para dor, em percentual menor (1,4%).

Acrescenta-se que as principais etiologias relacionadas à dor elencadas para os DE foram: trauma relacionado a procedimento invasivo e/ou lesão cirúrgica (57,3%); evolução da doença (20%); mobilidade prejudicada (7,6%); alteração vascular (4,4%); agentes lesivos biológicos, químicos, físicos e psicológicos (7,6%); sintomas da doença ou dor (4,2%); mobilidade física prejudicada (3,6%). Destaca-se que o valor “dor” esteve presente tanto como DE quanto como etiologia de um determinado diagnóstico, por exemplo, mobilidade física prejudicada. Porém, foram considerados para mineração dos dados os atributos prescritos nos cuidados de enfermagem, a fim de avaliar quais DE possuíam cuidados para dor prescritos com maior frequência à população de pessoas idosas com 75 anos ou mais.

Quando estratificadas por DE, as etiologias mostraram distribuições bastantes distintas entre eles. O DE Dor Aguda apresentou como principais etiologias o trauma relacionado ao procedimento invasivo e/ou lesão cirúrgica, evolução da doença e alteração vascular. Já o DE Dor Crônica apresentou como principais etiologias a evolução da doença e o processo inflamatório. Enfim, o DE Risco de Quedas, quando teve prescritos cuidados para dor, apresentou como principal etiologia a mobilidade prejudicada.

 Com efeito, foram identificados 60 atributos relacionados à dor em pessoas idosas vulneráveis, em diferentes tabelas do banco de dados, e os principais atributos em número de observações estão descritos na Figura 2 . Os atributos identificados foram organizados em sete grupos: (1) dor atual; (2) instrumentos e características de avaliação da dor; (3) título dos Diagnósticos de Enfermagem; (4) etiologia dos Diagnósticos de Enfermagem; (5) intervenções para alívio da dor; (6) consultorias a especialidades; (7) reavaliação da dor. A organização em grupos serviu de suporte para construção das definições conceituais dos grupos de atributos, aprimorando a compreensão semântica do MI desenvolvido. 

**Figura 2 -:** Principais atributos encontrados quanto ao gerenciamento da dor em pessoas idosas vulneráveis nos registros eletrônicos em saúde

Estes sete diferentes grupos de atributos foram agrupados e organizados em painéis de classificação: (1) Painel de Avaliação; (2) Painel de Intervenções; (3) Painel de Diagnósticos de Enfermagem e seus componentes; (4) Painel de Reavaliação.

 Assim sendo, a classificação dos atributos utilizou como base um MI de gerenciamento da dor validado ^(^ 2018 ^)^ , acrescentando em sua construção aspectos relacionados à cultura institucional e ao fluxograma de organização do sistema de informação hospitalar. Portanto, a partir dos RES organizou-se os componentes relacionados ao gerenciamento da dor em pessoas idosas vulneráveis. Para compreensão semântica do MI desenvolvido, a definição conceitual da classificação e dos grupos de atributos está descrita na Figura 3 . 

 Por fim, baseado nos dados de RES relacionados à dor que foram minerados na amostra do estudo, e após a classificação e organização destes atributos, foi desenvolvido um MI do gerenciamento da dor em pessoas idosas vulneráveis em um hospital universitário do sul do país. O MI desenvolvido é representado em uma estrutura visual na Figura 4 . 

**Figura 3 -:** Definição descritiva dos grupos de atributos e da classificação (painéis) utilizados no desenvolvimento do Modelo de Informação

## Discussão

A partir dos RES estruturados das pessoas idosas acima de 75 anos, foi desenvolvido o primeiro MI sobre o manejo da dor nesse grupo populacional. Apesar do banco de dados se restringir apenas a dados estruturados, a mineração com técnicas de ciência de dados permitiu obter informações amplas e com um volume significativo da amostra em estudo.

 Outros MI quanto ao gerenciamento da dor já foram desenvolvidos anteriormente com grandes bancos de dados nos Estados Unidos ^(^ 2017 ^)^ , e também no Brasil ^(^ 2021 ^)^ , porém não especificamente para pessoas idosas. O MI sobre o gerenciamento da dor norte-americano ^(^ 2017 ^)^ , com dados de diversas instituições de saúde, foi validado pela avaliação de especialistas ^(^ 2018 ^)^ , melhorando sua aplicabilidade clínica e oferecendo perspectivas para interoperabilidade entre sistemas de saúde. No entanto, o MI desenvolvido no presente estudo foi desenvolvido com os dados de uma única instituição hospitalar, o que reduz a sua capacidade de generalização. 

 A estratificação de idade entre pacientes idosos e idosos vulneráveis – com 75 anos ou mais - é uma ferramenta de operacionalização do acompanhamento da pessoa idosa hospitalizada na instituição ^(^ 2019 ^)^ . Entretanto, é controverso na literatura quanto à relevância da idade na prevalência da dor ^(^ 2018 ^)^ . A idade não foi observada como um fator significativamente diferente entre idosos com e sem dor no corpo ^(^ 2021 ^)^ , em dor no joelho ^(^ 2020 ^-^ 2023 ^)^ . Contudo, a idade é fator significante na intensidade da dor em pacientes com dor lombar ^(^ 2023 ^)^ , pós artroplastia total de quadril ^(^ 2022 ^)^ e em sinais de alterações da percepção dolorosa após acidente vascular encefálico ^(^ 2022 ^)^ . 

 Tais cenários evidenciam a complexidade da dor na pessoa idosa, justificando o desenvolvimento de MI que avaliem os cuidados relacionados ao gerenciamento da dor em grupos específicos. O MI desenvolvido neste estudo permitiu classificar os atributos minerados para a dor em avaliação, intervenções e reavaliação da dor. Esses quatro grandes painéis de classificação do gerenciamento da dor na instituição foram originários dos sete grupos de atributos relacionados à dor, baseados no MI estadunidense validado ^(^ 2017 ^)^ . 

 Salienta-se ainda que observa-se nos RES o uso de instrumentos unidimensionais na avaliação da dor ^(^ 2019 ^)^ , que permitem apenas a avaliação da intensidade, como as escalas analógica visual, numérica verbal e categórica verbal. Em geral, escalas unidimensionais são bons instrumentos na avaliação da dor e facilmente utilizáveis na prática clínica ^(^ 2018 ^-^ 2018 ^)^ , porém em pessoas idosas essas ferramentas podem não ser suficientes devido a incapacidades cognitivas, dificuldades de comunicação da experiência dolorosa e fatores comportamentais ^(^ 2018 ^,^ 2019 ^)^ . 

 Vale ressaltar que instrumentos de avaliação multidimensional da dor permitem melhor conhecimento sobre os aspectos psicológicos, sociais e econômicos que impactam a experiência dolorosa e também oferecem suporte a uma anamnese mais robusta da pessoa idosa ^(^ 2019 ^)^ . Existem cinco instrumentos multidimensionais de avaliação da dor validados no Brasil ^(^ 2016 ^-^ 2006 ^)^ , sendo alguns deles adaptados para pessoas idosas com capacidade limitada de comunicação ou para pacientes não comunicativos ^(^ 2016 ^-^ 2012 ^)^ , além de instrumentos específicos para pessoas idosas com demência ^(^ 2016 ^)^ . Nenhum destes instrumentos é utilizado na instituição, o que consiste em uma fragilidade no cuidado integral à saúde da pessoa idosa. 

 Observou-se nos RES uma variabilidade maior nos cuidados relacionados à dor prescritos por enfermeiros, englobando aspectos fisiológicos, psicológicos e sociais implicados no gerenciamento da dor. Apesar disso, o volume de observações indica uma maior importância dada à avaliação e às intervenções farmacológicas para dor, similar ao que é encontrado em outro estudo brasileiro ^(^ 2020 ^)^ . MI oferecem um suporte nesse sentido, permitindo que se compreendam as relações e as complexidades do fluxo de informação de temas de interesse para a prática de enfermagem ^(^ 2021 ^,^ 2017 ^)^ . 

**Figura 4 -:** Modelo de Informação do gerenciamento da dor em pessoas idosas com 75 anos ou mais

Na construção do MI, decidiu-se pela inclusão dos DE e seus componentes como um dos grupos representados devido ao grande número de registros estruturados, que representam a valorização institucional quanto à aplicação do processo de enfermagem. Foram incluídos no MI desenvolvido a presença de três DE com cuidados prescritos para dor, além daqueles que são específicos (DE Dor Aguda e DE Dor Crônica), sendo eles: Risco de Quedas, Conforto Prejudicado e Mobilidade Física Prejudicada.

 Esses três DE remetem ao prejuízo à funcionalidade motora e à mobilidade física, eventos comuns na pessoa idosa, principalmente nos hospitalizados ^(^ 2021 ^-^ 2021 ^)^ . É importante destacar que enfermeiros diagnosticam problemas de saúde e condições de risco sobre uma resposta humana que pode ou não estar associada a outras condições médicas ^(^ 2021 ^)^ . Assim sendo, o prejuízo da mobilidade que leva o enfermeiro a elencar os DE Risco de Quedas, Conforto Prejudicado ou Mobilidade Física Prejudicada identificam este sinal como uma necessidade de cuidado naquele momento. Uma vez que a respostas humanas são dinâmicas, os DE também o são, refletindo uma necessidade no momento que foi avaliado, podendo ter iniciado com essa demanda antes ou durante a hospitalização. 

 Visto que para a elaboração de nosso MI do gerenciamento da dor mineramos valores presentes nos cuidados prescritos, os dados encontrados nesta análise sugerem que sinais e sintomas de dor podem estar associados com um risco maior de ocorrência de quedas com a pessoa idosa vulnerável, considerando a recorrência de cuidados para dor prescritos no DE Risco de Quedas – o terceiro DE com maior volume de cuidados prescritos para dor. Esta característica não está presente no MI sobre gerenciamento da dor brasileiro desenvolvido com pacientes adultos ^(^ 2021 ^)^ . 

 Além disso, diferentes estudos ^(^ 2021 ^-^ 2021 ^)^ apontam que o risco de quedas não foi encontrado como uma variável associada à dor, independente da intensidade dela; ou como um fator protetor contra a ocorrência de quedas ^(^ 2020 ^)^ . Em contrapartida, um estudo realizado com adultos de meia idade e com pessoas idosas identificou a presença de sinais de dor como fator que aumenta risco para quedas ^(^ 2020 ^)^ , assim como o estudo ^(^ 2021 ^)^ realizado em pessoas idosas com 70 anos ou mais. Devido à característica descritiva do MI desenvolvido, não é possível afirmar se há uma associação entre a dor e o risco para quedas, porém, análises mais detalhadas dessa relação consistem em uma lacuna a ser melhor explorada. 

 Há fortes indícios de que uma menor rede de apoio à pessoa idosa esteja relacionada tanto ao risco de quedas, quanto ao pior manejo da dor ^(^ 2022 ^)^ , além de ser um fator relacionado à capacidade funcional nessa população ^(^ 2020 ^)^ . Isso, por si só, justifica protocolos de triagem e acompanhamento destes pacientes que devem ser avaliados de forma multidimensional, visando à organização da rede de apoio no planejamento de sua alta. 

O presente estudo apresentou limitações em seu desenho que precisam ser consideradas ao se avaliar os resultados apresentados. O MI desenvolvido contou apenas com dados estruturados dos prontuários eletrônicos dos pacientes para sua construção, não sendo incluídos documentos considerados importantes, como evolução e anamnese, além de exame físico de enfermagem, por se tratarem de registros em textos livres.

Por tratar-se de um estudo com utilização de registros secundários, também está sujeito a viés de informação, como erro de registros ou ausência de dados, como a data de internação dos pacientes, utilizada para determinar posteriormente a idade da amostra. Para mitigar esta limitação, a variável foi tratada de forma a considerar como data de internação dos pacientes a primeira ocorrência de prescrição de enfermagem ou a prescrição médica registrada.

Outra limitação importante foi o mapeamento por meio da análise exploratória e construção dos comandos de programação apenas por um pesquisador, que está sujeito a erros na extração de informações do banco de dados. Além disso, os atributos sobre o gerenciamento da dor em pessoas idosas vulneráveis foram mapeados em uma única instituição e em prontuários de pacientes internados em unidades clínicas e cirúrgicas.

 Quanto aos aspectos gerenciais e de pesquisa em saúde, os MI são parte inicial da construção de modelos semânticos que baseiam projetos de interfaceamento de sistemas de informação em saúde. Tanto o MI desenvolvido neste estudo quanto o MI de gerenciamento da dor em adultos desenvolvido no Brasil ^(^ 2021 ^)^ foram construídos a partir de dados de uma única instituição. Em propostas futuras, podem servir de suporte para a interoperabilidade de dados entre mais de uma instituição, colaborando para a construção de uma assistência orientada por dados e com potencial de generalização muito maior. 

## Conclusão

Em síntese, o MI permitiu obter um panorama da realidade assistencial do gerenciamento da dor em pessoas idosas hospitalizadas com 75 anos ou mais. Logo, o desenvolvimento do MI permitiu organizar os registros relacionados ao gerenciamento da dor, classificando-os em grandes grupos que refletem os cuidados prestados à pessoa idosa, assim como os recursos institucionais utilizados no gerenciamento da dor nesta população.

Observa-se uma maturidade institucional quanto à aplicação do processo de enfermagem de forma estruturada, além da presença de diferentes tipos de intervenções de enfermagem que englobam aspectos biológicos, psicológicos e psicossociais do cuidado de enfermagem.

Quanto às lacunas assistenciais observadas, identifica-se a ausência nos registros de instrumentos multidimensionais para gerenciamento da dor, o que dificulta a identificação deste sinal em pessoas idosas com prejuízo funcional ou déficit para compreender e comunicar a dor por meio de escalas numéricas.

O estudo permitiu observar que o prejuízo da mobilidade física aparece como um fator prevalente nos registros de cuidados para dor prescritos para pessoas idosas vulneráveis. Deduz-se que a presença de dor aliada ao prejuízo da mobilidade na pessoa idosa possa aumentar o risco de episódios de quedas. Entretanto, estudos com delineamentos mais robustos que permitam avaliar esta associação precisariam ser conduzidos.

Por fim, salienta-se que o MI desenvolvido não foi validado. Em um cenário prospectivo, a validação e a implementação do MI podem servir como suporte para a interoperabilidade semântica entre sistemas de informação ou no desenvolvimento de modelos preditivos sobre o manejo da dor, especialmente sobre a população idosa, além da otimização dos sistemas de informação hospitalares.
